# Ruthenium coordinated nanohybrids modulate tumor microenvironment and potentiate amplified phototherapy augmented immunotherapy of hypoxic tumor

**DOI:** 10.1016/j.mtbio.2025.102564

**Published:** 2025-11-17

**Authors:** Jingyao Li, Wenzhi Zhu, Qibao Zheng, Huixi Yi, Liyou Guo, Zhixiong Zhan, Nannan Fu, Muhammad Rizwan Younis, Chengzhi Jin, Junqiu Zhai, Dong-Yang Zhang

**Affiliations:** a Guangzhou Municipal and Guangdong Provincial Key Laboratory of Molecular Target & Clinical Pharmacology, The NMPA and State Key Laboratory of Respiratory Disease, The Fifth Affiliated Hospital and School of Pharmaceutical Sciences, Guangzhou Medical University, Guangzhou, 511436, China; b School of Pharmaceutical Sciences, Guangzhou University of Chinese Medicine, Guangzhou, 510006, China; c Department of Chemical and Biomolecular Engineering, University of California - Los Angeles, Los Angeles, CA, 90095, United States

**Keywords:** Hypoxia, Photodynamic therapy, Photothermal therapy, Immunotherapy, Tumor microenvironment, Ruthenium

## Abstract

Hypoxic tumor microenvironment (TME) greatly limits the efficacy of photodynamic therapy (PDT) and immunotherapy. Additionally, small molecule phototherapeutic agents not only require delivery vectors due to their poor water solubility, but also suffer from poor photostability. Herein, we present a synergy of nanocatalysts-amplified PDT, sustained photothermal therapy (PTT) and robust immunotherapy for eliminating hypoxic solid tumor and preventing distant tumor metastasis by constructing the ruthenium coordinated nanohybrids (defined as RuIP) consisting of ruthenium (Ru) ions, phototherapeutic agent (IR825), and polyvinylpyrrolidone (PVP). The coordination driven self-assembly of IR825 with Ru improves the photostability of IR825 and endows it i) with high catalytic O_2_ generation to alleviate tumor hypoxia and ii) antioxidant GSH depletion to promote PDT against tumor. Meanwhile, the coordination effect also enhances the photothermal conversion efficiency of IR825, especially the photothermal stability for sustained antitumor PTT. Under laser activation, self-assembled RuIP nanohybrid triggers the substantial killing of primary cancer in 4T1-bearing mice model through photodynamic and photothermal pathways, resulting in the induction of immunogenic cells death and the activation of antitumor immunity. Additionally, the catalytic O_2_ generation by RuIP alleviates tumor hypoxia and modulates the hypoxia-induced immunosuppressive TME, which further downregulates the PD-L1 expression in tumor cells, re-programs macrophage phenotype, and recruits more killer immune cells. The resulting immune memory successfully suppresses distant tumors, and restrict the growth of metastatic hypoxic tumor. Therefore, RuIP nanohybrid acts as an enzyme analogue to improve the efficiency of photo-immunotherapy, providing a translatable therapeutic strategy for hypoxic tumors.

## Graphical abstract

Ruthenium coordinated phototherapeutic nanohybrids were constructed via a self-assembly process for enhanced photo-immunotherapy of breast cancer by activating robust anti-tumor immunity and reversing immunosuppressive tumor microenvironment.

**Figure:**
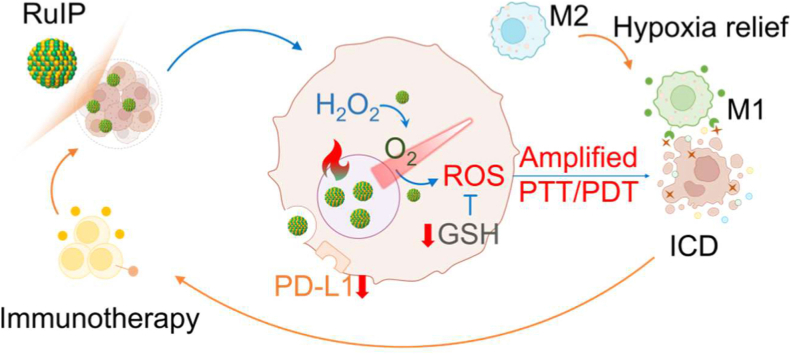

## 1 Introduction

Cancer immunotherapy is an emerging clinical treatment strategy, which holds great promise to prevent cancer metastasis and recurrence by activating the immune system of the body and improves patient's lifespan [[1], [2], [3]]. Although different immunotherapeutic approaches such as immune checkpoint blockade (ICB) therapy, chimeric antigen receptor T-cell therapy, and cancer vaccines have been developed and successfully applied in clinics [4,5], the prominent bottleneck is the immunosuppressive tumor microenvironment (TME) that leads to low cancer immunogenicity and inadequate infiltration of T cells, resulting in minimal immune response rate in only a small subset (∼10–30 %) of patients and poor treatment efficacy against hypoxic solid tumor [[6], [7], [8]]. Additionally, the available immunological medicines suffer from several limitations, including short half-life, off-target toxicity, and overpriced [9].

Tumor hypoxia, a prominent hallmark of solid tumor, is caused by a limited supply of oxygen (O_2_) and nutrients to the rapidly growing tumor due to the abnormal and leaky vasculature [10,11]. Such an inadequate O_2_ supply leads to an imbalance between the supply and demand of O_2_, resulting in tumor hypoxia that promotes the expression of hypoxia inducible factor-1α (HIF-1α), establishes immunosuppressive TME, and limits the therapeutic efficacy of O_2_ consumption therapy as well as immunotherapy [[12], [13], [14]]. For example, HIF-1α directly mediates the expression of tumor cell programmed death ligand-1 (PD-L1), which promotes the recruitment and aggregation of immunosuppressive regulatory T cells in solid tumors [15,16]. Besides, M2-type tumor-associated macrophages (TAMs) secrete inhibitory cytokines under tumor hypoxic conditions, such as transforming growth factor-β and interleukin-10 (IL-10), which together constitute an inhibitory immune TME to block the entry of killer immune cells [17,18].

Phototherapy, including photodynamic therapy (PDT) and photothermal therapy (PTT) is a minimally invasive cancer treatment strategy, which kills tumor cells by either producing reactive oxygen species (ROS) during PDT or through thermal ablation (PTT) with great tunability and spatiotemporal precision [19]. In addition, phototherapy effectively reverses immune suppression and augment immunotherapy by inducing immunogenic cell death (ICD) and activating the body's antitumor immune response [[20], [21], [22]]. However, since PDT depends on endogenous O_2_ to produce ROS and affected by elevated levels of antioxidant glutathione (GSH) in tumor cells [23,24], the overall performance of phototherapy is greatly compromised in hypoxic TME. Although, a number of studies have been reported to elevate endogenous O_2_ concentration through different strategies, such as hyperbaric oxygen therapy [25], nanocarrier O_2_ delivery [26], and reducing O_2_ consumption [27], the elevation of intratumoral O_2_ concentration is highly desired to alleviate tumor hypoxia and boost phototherapy [28,29], reverses immune suppression, improves the efficacy of immunotherapy, and avoid adverse systemic effects.

Self-assembly is believed to be a promising and facile method to build robust tumor phototherapeutic platforms, which can solve the problems of poor water solubility and poor photostability of phototherapeutic agent as well as self-toxicity of drug carriers [[30], [31], [32], [33]]. For instance, Song group reported the coordination-driven self-assembly of cyanine dyes with Cu^2+^. The fabricated Cy-Cu aggregates indicated enhanced photostability and favorable tumor phototherapy efficacy [32]. Recently, nanozymes have attracted great attention due to their multi-enzyme mimetic features, enabling them to alleviate oxidative stress and tumor hypoxia to modulate TME and achieve high treatment efficacy in anti-oxidant/anti-inflammatory therapy as well as tumor therapy [[34], [35], [36], [37]]. In our previous works, we demonstrated that self-assembled ultrasmall nanozymes can effectively decrease oxidative stress and inflammation, resulting in the management of acute kidney injury [38]. Inspired by our previous work, we anticipate that alleviating tumor hypoxia using self-assembled nanozyme coordinated with phototherapeutic drug would be an ideal strategy to reverse immune suppression and boost phototherapy augmented immunotherapy.

**Scheme 1:**
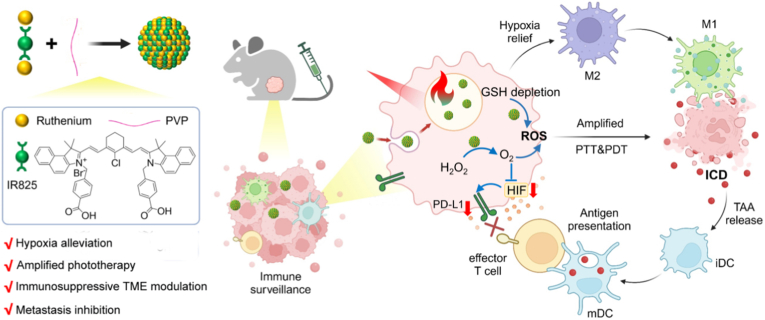
Schematic representation of self-assembled ruthenium coordinated nanohybrid (RuIP) for alleviating tumor hypoxia through catalytic O_2_ generation, modulating immune suppressive TME, and boosting phototherapy augmented immunotherapy to eliminate primary hypoxic solid tumor and further restrict cancer recurrence and metastasis.

Ruthenium complexes have emerged as a promising next-generation metal drugs with a potential characteristics of high antitumor activity, low toxicity, easy absorption, and quick excretion in the body [39,40]. Though ruthenium-based nanozymes have been developed for tumor catalytic therapy [41,42], ruthenium-coordinated phototherapeutic nanodrug is rarely reported. Herein, we develop a facile one pot self-assembly method to develop ruthenium coordinated nanohybrids (RuIP) constituted of ruthenium ion, phototherapeutic drug (IR825) and polyvinylpyrrolidone for modulating hypoxic TME and treating hypoxic solid tumor, as shown in Scheme 1. The coordination self-assembly strategy presents a high drug loading rate without the risk of any systemic toxicity associated with a nanocarrier. The obtained RuIP nanohybrid greatly enhances the photothermal performance of free IR825 under laser irradiation via coordination. Besides, RuIP nanohybrids effectively triggeres GSH depletion and catalytic oxygen generation to enhance the efficacy of PDT in hypoxic TME. Moreover, the ICD induced by phototherapy and reversed immunosuppressive TME through alleviating tumor hypoxia down-regulate the expression of PD-L1, re-program macrophage phenotype, and recruit more immune cells to achieve enhanced immunotherapy that further restrict distant tumor metastasis. This ruthenium coordinated nanohybrids integrated with photo- and immuno-synergetic therapy through TME modulation have great promise to precisely eliminate hypoxic solid tumors with fewer adverse effects.

## 2 Experimental section

### 2.1 Materials

Ruthenium trichloride hydrate (RuCl_3_·xH_2_O, 35.0–42.0 % Ru basis), 5,5′-dithio bis-(2-nitrobenzoic acid) (DTNB, 98 %), 1,3-diphenylisobenzofuran (DPBF, 97 %), and thiazolyl blue (MTT, 98 %) were purchased from Aladdin Reagent (China). IR825 (97 %), GSH (98 %) and tris(4,7-diphenyl-1,10-phenanthroline)ruthenium(II) dichloride complex (Ru(dpp)_3_]Cl_2_, 95 %) were bought from Macklin Biochemical Technology Co., Ltd. (China). Polyvinylpyrrolidone (PVP, average M_w_ ∼55,000) and propidium iodide (PI)/calcein acetoxymethyl ester (calcein-AM) assay kit were obtained from Sigma-Aldrich (USA). Hydrogen peroxide (H_2_O_2_, AR, 30 wt%) was bought from Sinopharm Group Chemical Reagent Co. LTD. (China). 2′,7′-dichlorodihydrofluorescein diacetate (DCFH), apoptosis detection kit (PI/Annexin V-FITC), and lipopolysaccharide (LPS) were acquired from Beyotime Biotechnology CO. LTD. (China). HIF-1α, high mobility group protein B1 (HMGB1), calreticulin (CRT), glyceraldehyde-3-phosphate dehydrogenase (GAPDH) antibodies were purchased from Signalway Antibody (USA) and Cell Signaling Technology (USA). Mouse IL-10 and IL-12 enzyme-linked immunosorbent assay (ELISA) kit were obtained from Wuhan Boster Biological Technology Co. Ltd. Deionized (DI) water (Millipore, USA) was employed in all experiments.

### 2.2 Fabrication of RuIP

RuCl_3_·xH_2_O (18.67 mg) and PVP (15 mg) were dissolved in 72 mL of DI water at 25 °C under vigorous stirring. After 30 min, IR825 (5.4 mg) in 8 mL of dimethylsulfoxide (DMSO, AR) was slowly added to the above mixed solution under continued stirring for next 24 h (h). Then the mixture was centrifuged by ultrafiltration (MWCO: 30 kDa, 4000 rpm, 10 min) and washed 3 times with water to obtain the self-assembled RuIP nanohybrids. The mass proportion of IR825 in the RuIP was measured using UV–vis spectrophotometer. Simultaneously, the content of Ru element in the RuIP was determined by inductively coupled plasma mass spectrometry (ICP-MS). The mass fraction of PVP in the RuIP was calculated through subtracting the mass fraction of Ru element and IR825.

### 2.3 Characterization of RuIP

The morphological characterization and elemental mapping of RuIP nanohybrids were done by transmission electron microscopy (TEM, JEOL, JED-2300T, Japan) with an energy spectrometer (JEOL, JEM-F200, Japan). The hydrodynamic diameter and zeta potential were determined via a granulometer (Malvern, Nano-ZS ZEN 3600, England). The X-ray photoelectron (XPS) spectra were acquired on an X-ray photoelectron spectrometer (Thermo scientific k-alpha, USA). The absorption spectra of all samples were recorded by a Cary 5000 UV–vis spectrophotometer (Agilent, USA), while the fluorescence was measured by a fluorescence spectrometer (DUETTA, Japan‌).

### 2.4 Photothermal measurements

Concentration-dependent (0–100 μg/mL) photothermal activity of RuIP was recorded after exposing RuIP solutions to 808 nm laser irradiation (1 W/cm^2^) for 5 min. An infrared thermal imaging camera (FLIR A308, Sweden) was used to record real-time temperature elevation. Then, the RuIP solution (100 μg/mL) was irradiated by an 808 nm laser at a power density of 1 W/cm^2^ for 5 min, and then cooled to room temperature. Next, the photothermal conversion efficiency (PCE) of RuIP was calculated following the reported method [43]. Further, the photothermal stability of RuIP (100 μg/mL) and IR825 (20 μg/mL) were assessed under four heating (1 W/cm^2^, 5 min) and cooling cycles. The absorption spectra of RuIP and IR825 before and after 20 min of laser irradiation were recorded.

### 2.5 Measurement of ROS generation

The ROS generation ability of RuIP under laser illumination was evaluated using the DBPF probe. Briefly, the control, IR825 and RuIP solutions containing DPBF (40 μM) were irradiated every 15 s for 90 s with an 808 nm laser at a power density of 1 W/cm^2^. The absorption spectra and optical density (OD) of DPBF at 410 nm were recorded using an UV–vis spectrophotometer.

### 2.6 Evaluation of catalytic activity and O_2_ generation

The dissolved O_2_ content of H_2_O_2_ solution (1 M) containing different concentrations (0–20 μg/mL) of RuIP was measured every 30 s for 5 min using a dissolved O_2_ analyzer. Furthermore, the kinetic analysis of RuIP with catalase (CAT)-like activity was conducted at 25 °C in phosphate buffer solution (PBS, pH 7.4). The RuIP (20 μg/mL) was added to varied concentrations of H_2_O_2_ solution (final concentration: 5–15 mM), and then the absorption at 240 nm were monitored using UV–vis spectrophotometer. The kinetic parameters were calculated by using Michaeli-Menten equation.

### 2.7 Evaluation of GSH depletion

A GSH specific probe DTNB was acted as an indicator to assess the GSH depletion capacity of RuIP. GSH solution (1 mM, 60 μL) was mixed with different concentrations of RuIP (0–50 μg/mL, 540 μL) for 4 h at 37 °C in a shaker. The above solutions were underwent ultrafiltration (MWCO: 30 kDa, 4000 rpm, 10 min). Then, DTNB solution (2 mM, 140 μL) was incubated with filtrates (460 μL) for 30 s. Next, the absorption spectra were recorded using the UV–vis spectrometer.

### 2.8 *In vitro* therapeutic evaluation

MTT assay: 4T1 and human embryonic kidney (HEK) 239T cells (5 × 10^3^ cells/well) were cultured into 96-well plates overnight. Then, the medium was replaced with fresh medium (100 μL) containing various concentrations of RuIP (0–100 μg/mL) or IR825 (0–20 μg/mL). After 24 h, MTT (20 μL/well, 5 mg/mL) was added and incubated for 4 h followed by the addition of DMSO (150 μL). Then, the absorbance of MTT reagent at 490 nm was measured by a microplate reader (Tecan Infinite® M Nano, Switzerland). For hypoxic evaluation, the O_2_ concentration was set to 1 % in the hypoxic group and 21 % in the normoxic group. The L-treated groups were exposed to light (808 nm, 1 W/cm^2^, 5 min) after 12 h of drug treatment.

Calcein-AM/PI assay: 4T1 cells (2 × 10^5^ cells/well) were cultured into 6-well plates under hypoxic conditions for 24 h. Then, RuIP nanohybrids (100 μg/mL) or IR825 (20 μg/mL) was supplemented in fresh culture medium. After 4 h, the cells were treated with/without 808 laser (1 W/cm^2^) for 5 min. Next, the cells were co-stained with Calcein-AM/PI, followed by fluorescence imaging (Leica DMi8, Germany). Calcein-AM, excitation: 494 nm, emission: 517 nm; PI, excitation: 535 nm, emission: 617 nm.

Cellular apoptosis: 4T1 cells (2 × 10^5^ cells/well) were cultured into 6-well plates under hypoxic condition for 24 h. The cells were then divided into six groups: PBS, IR825 (20 μg/mL), RuIP (100 μg/mL), PBS + L, IR825+L, and RuIP + L. After 4 h, the cells were treated with/without 808 laser (1 W/cm^2^) for 5 min. The cells were then stained with Annexin V-FITC and PI, followed by flow cytometry (Beckman CytoFLEX S.4, USA).

Transwell assay: 4T1 cells (5 × 10^4^ cells) in serum-free medium (500 μL) were cultured in the upper chamber and 700 μL of medium was added to the lower chamber of 24-well Transwell® permeable supports (Corning, USA) under hypoxic conditions. The cells in the upper chamber were treated with different concentrations of RuIP (0–100 μg/mL) for 24 h, and the cells in the lower chamber were fixed with paraformaldehyde for 30 min. Subsequently, the cells were stained with crystal violet for 30 min, followed by imaging using a microscope (Echo Revolve, USA). The migration inhibition rate was quantified by using ImageJ software (NIH, USA).

Evaluating biocatalysis: 4T1 cells (1 × 10^5^ cells/dish) were cultured into confocal dishes under hypoxic or normoxic condition for 24 h. Then, the cells were treated with RuIP nanohybrids (50 μg/mL). After 24 h, the cells were stained with phosphorescent oxygen probe Ru(dpp)_3_]Cl_2_ (10 μM). Subsequently, the cells were imaged by a confocal laser scanning microscopy (CLSM, Zeiss LSM900, Germany). Ru(dpp)_3_]Cl_2_, excitation: 488 nm, emission: 625 nm; Hoechst, excitation: 405 nm, emission: 461 nm.

Assessing *in vitro* GSH depletion: 4T1 cells (2 × 10^5^ cells/well) were cultured into 6-well plates for overnight. Then, the cells were treated with RuIP nanohybrids (50 μg/mL). After 24 h, *in vitro* GSH content was measured by a GSH/GSSG detection kit according to the manufacturer's instructions (Beyotime Biotechnology CO. LTD., China).

Determining intracellular ROS level: 4T1 cells (1 × 10^5^ cells/dish) were cultured into confocal dishes for overnight. Then, they were divided into six groups: PBS, IR825 (20 μg/mL), RuIP (100 μg/mL), PBS + L, IR825+L, and RuIP + L. After 4 h, the cells were treated with/without 808 nm laser (1 W/cm^2^) for 5 min. Next, the cells were stained with a DCFH probe, followed by CLSM imaging. DCFH, excitation: 488 nm, emission: 525 nm.

### 2.9 *In vitro* immunotherapeutic evaluation

Western bloting: 4T1 cells (2 × 10^5^ cells/well) were seeded in 6-well plates and incubated for 24 h. After treated with various concentrations (0–100 μg/mL) of RuIP for 12 h, the cells were placed in a lysis buffer containing complete protease inhibitors. The samples were heated at 95 °C for 5 min and separated by sodium dodecyl sulfate (SDS) buffer. Next, the separated proteins were transferred to a poly(vinylidene fluoride) membrane, and blocked with 5 % skim milk powder at room temperature for 1.5 h. Subsequently, the membranes were incubated with HIF-1α (CST, 36169, USA) and glyceraldehyde-phosphate dehydrogenase (GAPDH, Servicebio, China) antibodies for overnight, and incubated with a goat anti-rabbit IgG (H&L)-HRP conjugated antibody at room temperature for 1.5 h. Finally, the ECL chemiluminescence kit (NCM Biotech, China) was used for visualization, and the protein quantitative analysis was performed by ImageJ software. The cell expression was standardized according to GAPDH protein.

CRT and HMGB1 expression: 4T1 cells (1 × 10^5^ cells/dish) were cultured into confocal dishes under hypoxic condition for 24 h. Then, they were divided into six groups: PBS, IR825 (20 μg/mL), RuIP (100 μg/mL), PBS + L, IR825+L, and RuIP + L. After 4 h, the cells were treated with/without 808 nm laser (1 W/cm^2^) for 5 min, followed by fixation with 4 % paraformaldehyde for 25 min at room temperature and infiltration with 0.1 % Triton X-100 for 10 min. After blocking with 1 % bovine serum albumin (BSA) for 1 h, the cells were incubated with CRT (abcam, ab92516, Britain) or HMGB1 (abcam, ab79823, Britain) antibodies at 4 °C overnight. Then, the cells were washed with PBS and then stained with goat anti-rabbit IgG (abcam, ab150077, Britain) for 1 h at room temperature. Finally, the cells were stained with Hoechst 33342 (Beyotime, China) and underwent CLSM imaging. Excitation: 488 nm, emission: 525 nm.

Evaluation of intracellular ATP level: 4T1 cells (2 × 10^5^ cells/well) were cultured into 6-well plates under hypoxic conditions for 24 h. Then, they were divided into six groups: PBS, IR825 (20 μg/mL), RuIP (100 μg/mL), PBS + L, IR825+L, and RuIP + L. After 4 h of incubation, the cells were treated with/without 808 nm laser (1 W/cm^2^) for 5 min. After 4 h, *in vitro* ATP content was measured according to the manufacturer's protocol (Beyotime, China).

PD-L1 expression: 4T1 cells (1 × 10^5^ cells/dish) were cultured into confocal dishes under hypoxic condition for 24 h. The cells were treated with RuIP at various concentrations (0–50 μg/mL). After 12 h of incubation, the cells were fixed with 4 % paraformaldehyde for 25 min at room temperature and filtered with 0.1 % Triton X-100 for 10 min. After blocking with 1 % BSA for 1 h, the cells were incubated with PD-L1 antibody (abcam, ab213480, Britain) at 4 °C overnight. Then, the cells were washed with PBS and then stained with goat anti-rabbit IgG (abcam, ab150077, Britain) for 1 h at room temperature. Next, the cells were stained with Hoechst 33342 (Beyotime, China) and underwent CLSM imaging. The quantitative analysis was performed using ImageJ software. Excitation: 488 nm, emission: 525 nm.

Evaluation of macrophage polarization: RAW264.7 cells (6 × 10^5^ cells/well) were seeded in 6-well plates and stimulated with IL-4 (Novoprotein, China, 100 ng/mL) or LPS (200 ng/mL) for 24 h to establish M2 and M1 macrophages, respectively. Then, the cells were treated with different concentrations of RuIP (0–50 μg/mL) or IR825 (0–10 μg/mL), followed by incubation with the F4/80 (BV421, BioLegend), CD86 (APC, BioLegend) and CD206 (FITC, BioLegend) antibodies for flow cytometric analysis. Meanwhile, the proteins were extracted for western blot analysis of iNOS (Affinity, AF0199, USA), a typical marker of M1 macrophages, and Arg-2 (Affinity, DF6657, USA), a typical marker of M2 macrophages. Then, the cytokine levels in cell supernatants were analyzed by IL-10 or IL-12 ELISA kits according to the manufacturer's protocol.

RAW 264.7 cells (1 × 10^5^ cells/dish) were cultured into confocal dishes for overnight. Then, the cells treated with different concentrations of RuIP (0–50 μg/mL) or IR825 (0–10 μg/mL). After 24 h, the cells were stained with a DCFH probe, followed by CLSM imaging.

Evaluation of dendritic cells (DC) maturation: Bone marrow-derived DCs (BMDCs) were acquired from BALB/c mice based on the prior method to construct the DC maturation protocol [15]. 4T1 cells (1 × 10^5^ cells/well) were seeded in 12-well plates and divided into six groups: PBS, IR825 (20 μg/mL), RuIP (100 μg/mL), PBS + L, IR825+L, and RuIP + L. After 4 h of incubation, the 4T1 cells were treated with/without an 808 nm laser (1 W/cm^2^) for 5 min. After 4 h, the cell supernatants from various treatment groups were added to BMDCs for 24 h, and then stained with the CD11c (PE-Cy7, BioLegend) and CD86 (APC, BioLegend) antibodies for flow cytometry analysis.

### 2.10 *In vivo* animal experiments

Female BALB/c mice aged 6–8 weeks were bought from Beijing Vital River Laboratory Animal Technology Co., Ltd. (China), and the animal experiment protocol (GY2024-538) was approved by Guangzhou Medical University (China). 2 × 10^6^ 4T1 cells dissolved in 90 μL PBS were injected into the right side of the mice, and 1 × 10^6^ 4T1 cells dissolved in 45 μL PBS were injected into the left side of the mice to establish the bilateral tumor mouse model. The short and long diameters of tumor tissues were recorded and the tumor volumes were calculated according to the formula: short diameter^2^ × long diameter/2.

### 2.11 *In vivo* biosafety

Hemolysis assay: Fresh red blood cells (RBCs) were obtained by collecting blood through the orbit and washed several times with PBS. RuIP solutions (100 μL, 12.5–4000 μg/mL) were added to 100 μL of RBCs suspensions and incubated for 8 h. After centrifugation (1000 rpm, 5 min), the OD value of collected supernatants were recorded at 541 nm. PBS and water were used as negative and positive controls, respectively. All solutions without RBCs were treated in the same way to deduct the effect of material background, and the hemolysis ratio was calculated according to the previous method [44].

*In vivo* biocompatibility: BALB/c mice received intravenous injection of RuIP (8 mg/kg) or equal volume of PBS, and the body weight of mice were recorded every 5 days for 30 days. After 30 days, the major organs and blood were collected. The major organs were stained with hematoxylin and eosin (H&E) and imaged by a microscope. While, the levels of liver/renal function biomarkers (BUN: blood urea nitrogen, CREA: creatinine, AST: aspartate aminotransferase, ALT: alanine aminotransferase) in serum were analyzed to determine systemic toxicity.

### 2.12 *In vivo* biodistribution

The tumor-bearing mice received intravenous injection of RuIP (8 mg/kg). After 24 h, all mice were sacrificed and the vital organs were collected. After digestion with chloroazotic acid, the distribution of RuIP in various tissues and tumors was analyzed by ICP-MS.

### 2.13 Bimodal photoacoustic/photothermal imaging

The tumor-bearing mice received intravenous injection of RuIP (8 mg/kg) or equal IR825 (1.6 mg/kg). The time-dependent *in vivo* photoacoustic signals were acquired through a PA imager.

The tumor-bearing mice received intravenous injection of RuIP (8 mg/kg), IR825 (1.6 mg/kg) or equal volume of PBS. After 2 h, the mice were irradiated by an 808 nm laser (1 W/cm^2^) for 5 min. The *in vivo* photothermal imaging of tumor region was recorded by a thermal imager.

### 2.14 *In vivo* photo/immunotherapy

When the subcutaneous tumor grew to approximately 100 mm^3^ on the right side (primary tumor) and 80 mm^3^ on the left side (distant tumor), the mice were assigned to five groups (n = 6): (i) PBS, (ii) L, (iii) RuIP, (iv) IR825+L, and (v) RuIP + L. The mice in groups (iii) and (v) received intravenous injection of RuIP (40 mg/kg), the mice in group (iv) received intravenous injection of IR825 (8 mg/kg), and the other groups were injected with PBS. At 2 h post-drug injection, the primary tumors from mice in groups (ii), (iv) and (v) received 808 nm laser (1 W/cm^2^, 5 min) irradiation. The tumor volume and body weight of all mice were measured every 2 days for 2-weeks. After 3 days, one mouse from each group was randomly selected to harvest the primary tumors for different analysis including, hematoxylin and eosin (H&E), TdT-mediated dUTP nick-end labeling (TUNEL), HIF-1α, CRT, HMGB1, and PD-L1 staining. The quantitative analysis of immunofluorescence staining images was performed using the ImageJ software. After 14 days, all mice were sacrificed and tumors, spleen, and blood were collected. The tumors were weighed and photographed. The levels of cytokines (IL-10 and IL-12) in serum were analyzed using corresponding ELISA kits (‌ELK Biotechnology, China).

### 2.15 Analysis of immune cells

The spleen and distant tumors collected from each group after 14 days treatment were underwent further immune analysis. Single cell suspensions were obtained by grinding spleen and passing through the membrane, and subsequently stained with DC (CD11c (PE-Cy7, BioLegend), CD86 (APC, BioLegend), and CD80 (FITC, BioLegend)) antibodies for flow cytometry analysis. Subsequently, the cells were stained with macrophage (CD11b (PE-Cy7, BioLegend), F4/80 (BV421, BioLegend), CD86 (APC, BioLegend), and CD206 (FITC, BioLegend)) and T lymphocyte (CD3 (PE, BioLegend), CD4 (FITC, BioLegend) and CD8 (PE-Cy7, BioLegend)) antibodies for flow cytometry analysis.

### 2.16 Statistical analysis

All the data were expressed as the mean ± standard deviation from independent replicates. Significance was acquired by Student's t -test by Origin95 software, ∗P < 0.05, ∗∗P < 0.01, and ∗∗∗P < 0.001.

## 3 Results and discussion

**Fig. 1:**
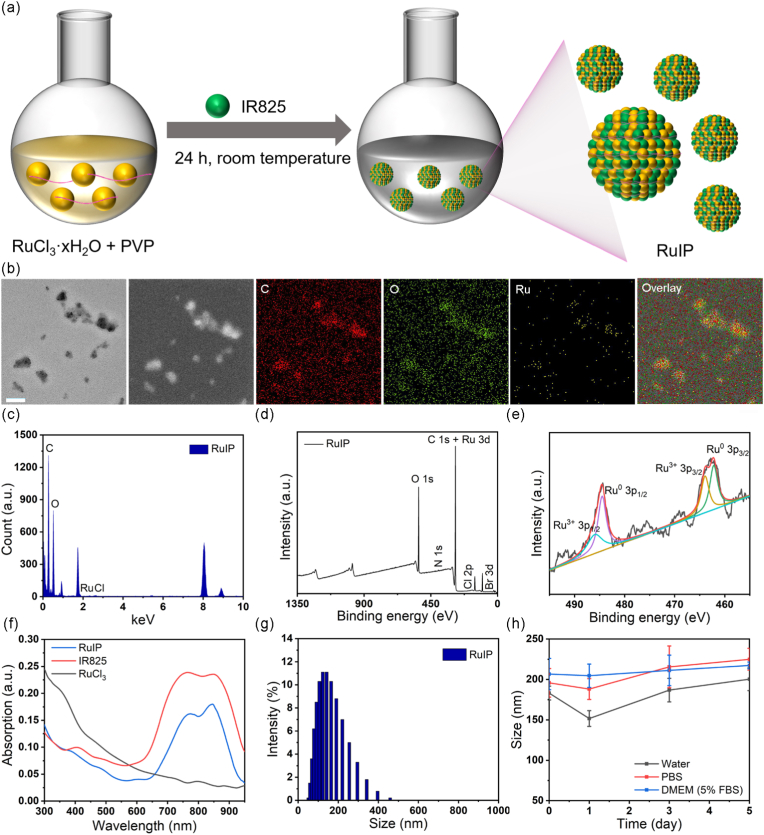
(a) Schematic illustration of the coordination of IR825 with Ru ions to form self-assembled RuIP nanohybrids. (b) TEM image and element mapping of RuIP nanohybrids. Scale bars: 200 nm. (c) Energy dispersive X-ray spectrum of RuIP nanohybrids. (d) Survey scan and (e) Ru *3p* XPS spectra of RuIP nanohybrids. (f) Absorption spectra of free IR825, RuCl_3_, and RuIP solutions. (g) Hydrodynamic diameter and zeta potential of RuIP nanohybrids as measured by dynamic light scattering. (h) Time-dependent physiological stability of RuIP in water, PBS and DMEM containing 5 % FBS.

The phototherapeutic agent IR825 with poor water solubility and favorable biocompatibility was coordinated with Ru ions and PVP through non-covalent interactions at a room temperature, resulting in the formation of self-assembled RuIP nanohybrids (Fig. 1a). Transmission electron microscopy (TEM) indicated the granular and spherical morphology of RuIP with an approximate diameters of 100 nm (Fig. 1b), suggesting preferential tumor enrichment because of enhanced permeability and retention (EPR) effects [45]. The energy dispersive (EDS) elemental mapping confirmed the uniform distribution of Ru, carbon, and oxygen elements in RuIP nanohybrids as shown in Fig. 1b–c, suggesting the successful formation of Ru ions coordinated IR825 (RuIP). X-ray photoelectron spectroscopy (XPS) analysis further confirmed the presence of all the characteristic peaks of Ru, carbon, nitrogen, chlorine, and bromine in the XPS spectrum of RuIP (Fig. 1d). Intriguingly, the two peaks of the valence states (0 and 3^+^) of ruthenium, Ru^0^ 3p_1/2_, Ru^3+^ 3p_1/2_, Ru^0^ 3p_3/2_, and Ru^3+^ 3p_3/2_, were found at 62.4 eV, 61.7 eV, 65.1 eV and 64.3 eV (Fig. 1e), suggesting the catalytic activity of Ru in RuIP [46]. UV–Vis analysis did not show any noticeable change in the absorption spectrum of IR825 and RuIP, suggesting the successful coordination of IR825 with Ru ions in RuIP (Fig. 1f). However, owing to the coordination interaction of IR825 with Ru ions, RuIP nanohybrids exhibited strong fluorescence at ∼850 nm as compared to IR825 (Fig. S1). The quantitative analysis confirmed 3 % (Ru): 20 % (IR825): 77 % (PVP) in RuIP as determined by ICP-MS (Fig. S2a–b). It has been noted that RuIP nanohybrids possessed negative surface zeta potential (−15.2 ± 3.2 mV) with a hydrodynamic diameter of about 170 nm (Figs. S3 and 1g) as suggested by dynamic light scattering (DLS). Whereas the hydrodynamic diameter of RuIP did not change even after incubation in water, PBS dulbecco's modified eagle medium (DMEM) containing 5 % fetal bovine serum (FBS) for 5 days (Fig. 1h), implying good physiological stability of RuIP nanohybrids.

**Fig. 2:**
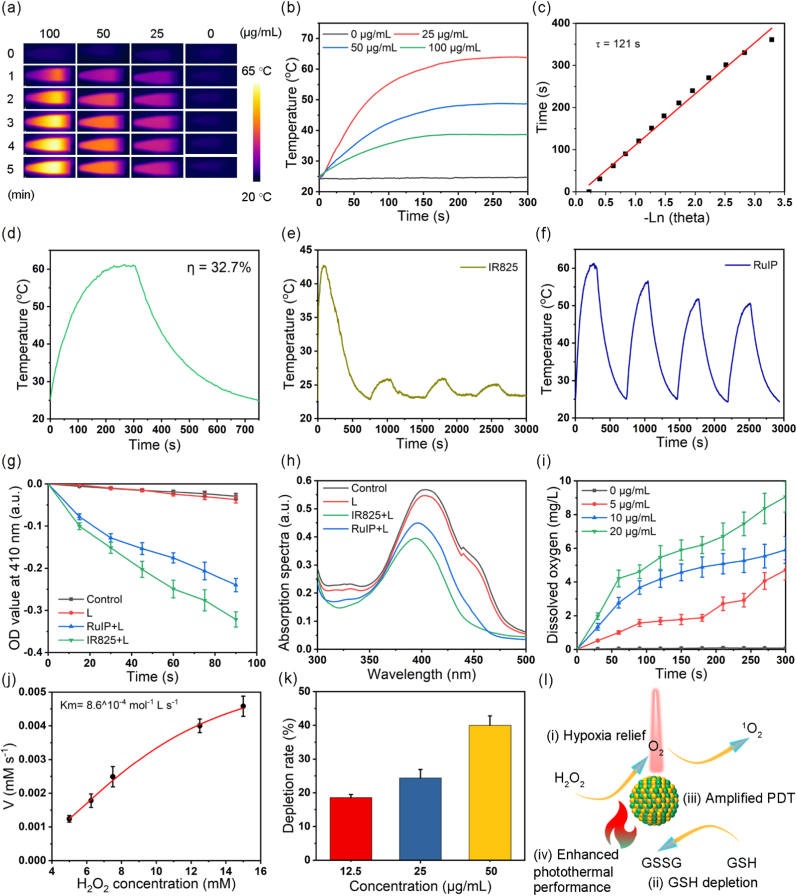
(a) Thermal imaging and (b) heating curves of RuIP solutions at different concentrations. (c) The linear relationship between cooling time and -lnθ. (d) The process of heating and cooling in RuIP solution to evaluate photothermal conversion efficiency. Photothermal stability of (e) IR825 and RuIP nanohybrids (f) with repeated illumination. (g) OD of DBPF at 410 nm under different treatments as indicated. (h) Absorption spectra of DBPF under different treatments as indicated. (i) Dissolved O_2_ content in solution containing H_2_O_2_ (0.3 wt%) and varied concentrations of RuIP. (j) Kinetics experiment for CAT-like activity of RuIP with different concentrations (5–15 mM) of H_2_O_2_. (k) Depletion rate of GSH by RuIP with indicated concentrations. (l) Schematic representation of the catalytic O_2_ generation, GSH depletion, singlet oxygen generation, and heat production under laser illumination mediated by RuIP nanohybrids.

Next, we determined the photo-responsive (photothermal and photodynamic) activity of RuIP nanohybrids. First, the photothermal performance was assessed under near infrared (NIR) laser irradiation. Fig. 2a–b showed the concentration-dependent photothermal activity of RuIP. Compared to the control group (ΔT: 0.48 °C), RuIP abruptly increased the solution temperature (ΔT: 39.2 °C) at a higher concentration (100 μg/mL) and exhibited superior conversion efficiency (η = 32.7 %) than free IR825 (23.0 %) and the commercial dye ICG (13.5 %, Fig. 2c–d) [47,48]. Moreover, IR825 showed poor photothermal stability under different laser on/off cycles as the temperature significantly dropped to 25.0 during 4th cycle of heating and cooling (Fig. 2e), which is attributed to the photobleaching effect. Although RuIP nanohybrids also displayed slight decrease in photothermal activity, the maximum temperature was remained ∼50.6 °C even at 4th cycle of heating and cooling (Fig. 2f), implying the good photothermal stability of RuIP. Complementally, the absorption spectrum of IR825 decreased distinctly after irradiation, while that of RuIP decreased only slightly (Fig. S4). Such an enhanced photostability of RuIP nanohybrids is attributed to the metal coordination effect.

Furthermore, the photodynamic properties of RuIP and free IR825 were investigated by using the ROS probe (DPBF). Compared to the control groups, both IR825 and RuIP displayed time-dependent decrease in DPBF absorption at 410 nm as shown in Fig. 2g, implying the production of ROS. It is notable to mention that owing to the metal coordination, RuIP showed less ROS production than free IR825 (Fig. 2h). Considering the presence of Ru ions in RuIP nanohybrids, we assessed the enzyme mimetic activity and O_2_ generation behavior of RuIP. The production of O_2_ was monitored by using a dissolved oxygen meter. There was no apparent O_2_ generation in H_2_O_2_ solution. In contrast, a significant O_2_ generation has been recorded in the presence of a higher concentration of RuIP and H_2_O_2_ solution (Fig. 2i), presenting concentration-dependent catalytic activity of RuIP as higher RuIP concentration resulted in more O_2_ bubbles in the solution (Fig. S5). Whereas, the kinetic analysis suggested 8.6^^^10^−4^ mol^−1^ L s^−1^ Michaelis constant (*K*_m_) of RuIP (Fig. 2j). Since GSH plays a crucial role in maintaining intracellular redox balance, which can react with ROS to reduce the antitumor effect of PDT, the GSH depletion capacity of RuIP was evaluated using GSH/GSSG detection assay kit. Approximately 40 % GSH was depleted by RuIP (50 μg/mL) as shown in Fig. 2k, which suggested good GSH depletion activity of RuIP to boost the effect of PDT. These findings verified that the RuIP nanohybrids is able to potentiate phototherapy through catalytic O_2_ generation and GSH depletion, simultaneously (Fig. 2l).

**Fig. 3:**
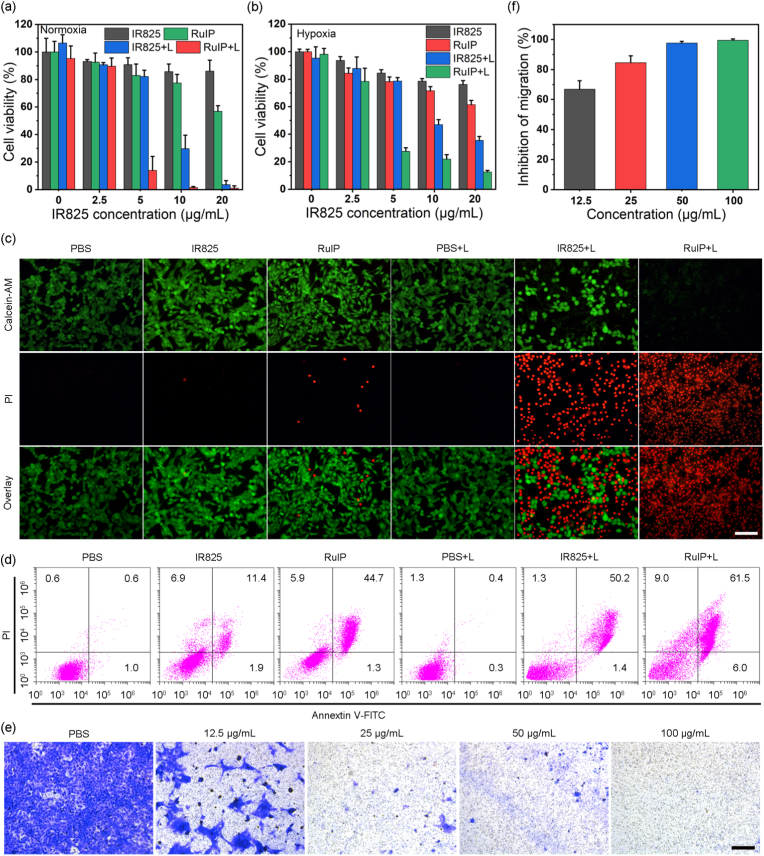
Cellular viability after treated with indicated groups under (a) normoxia or (b) hypoxia conditions. (c) Representative fluorescence images of 4T1 cells-treated with indicated groups in dead/live staining assay. Scale bar: 100 μm. (d) Apoptosis detection assay of 4T1 cells with indicated treatments as measured by flow cytometry. (e) Transwell assay to study the inhibition of cell migration after treated with different concentrations of RuIP and (f) Quantitative analysis of transwell assay. Scale bar: 100 μm.

Inspired by the improved photo-responsive properties of RuIP nanohybrids, the *in vitro* therapeutic efficacy was assessed using 4T1 cancer cells. First, the MTT assay was performed by incubating 4T1 cells at 1 % O_2_ and 21 % O_2_ to mimic hypoxic and normoxic environments, respectively. Although under dark conditions in normoxic environment, both free IR825 and RuIP showed negligible reduction in cellular viability (Fig. 3a), a substantial killing of 4T1 cancer cells were recorded by both the groups under NIR laser excitation. More than 95 % cells were killed by 100 μg/mL RuIP and 20 μg/mL IR825, indicating the good phototherapeutic antitumor effect under normoxic conditions. In contrast, under hypoxic conditions, IR825 showed significant decline in therapeutic efficacy as the number of viable cells were ∼40 %. Whereas, the number of viable cells were about 10 % in RuIP-treated group (Fig. 3b). Such a sustained therapeutic efficacy even under hypoxic conditions is attributed to the catalytic performance of RuIP to generate O_2_ in TME and alleviate hypoxic conditions, resulting in superior therapeutic efficacy than free IR825. Subsequently, Calcein-AM/PI live and dead assay was performed. The obtained results were in agreement with the MTT assay as the number of dead cells significantly increased in both IR825+L and RuIP + L groups (Fig. 3c). Complementarily, flow cytometery analysis indicated the greatest proportion of apoptotic (67.5 %) and necrotic (9.0 %) cells in the RuIP + L group as shown in Fig. 3d. Interestingly, at a concentration of 100 μg/mL, RuIP presented less than 20 % toxicity towards the normal (HEK293T) cells (Fig. S6). Since hypoxic TME plays a key role in tumor cell metastasis, we performed transwell and wound healing assays to study the inhibitory effect of RuIP on cancer cell migration. Transwell assay confirmed that RuIP nanohybrids effectively inhibited the migration of cancer cell in a concentration-dependent manner (Fig. 3e–f) as more than 90 % of cell migration was inhibited by 100 μg/mL RuIP. Whereas, ∼70 % cellular inhibition was recorded in wound healing assay (Figure S7-8). These results confirmed that RuIP nanohybrids can effectively relieve hypoxic conditions and potentiate phototherapy.

**Fig. 4:**
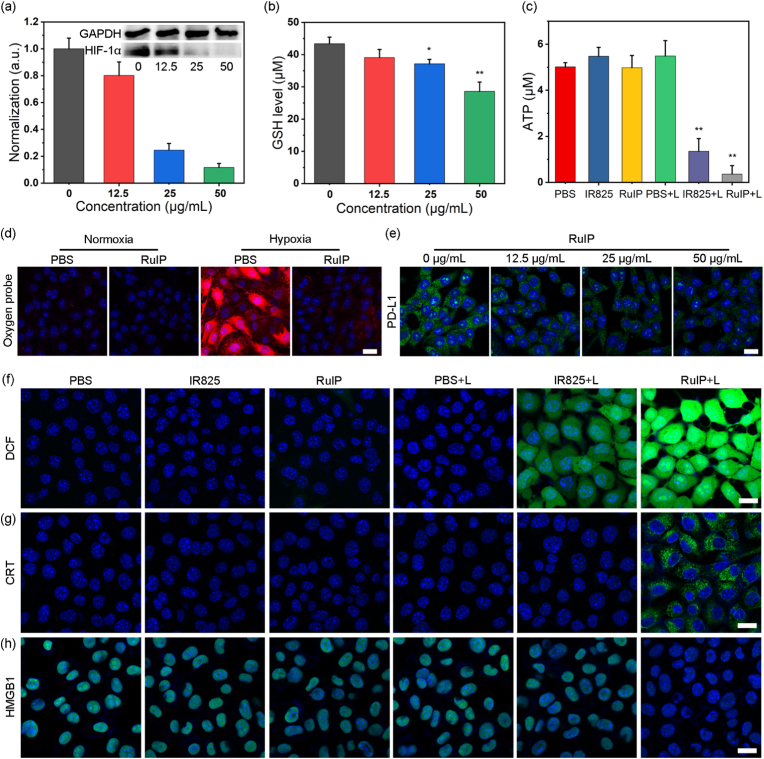
(a) Western blot analysis of HIF-1α expression in 4T1 cells after indicated treatments. (b) The GSH level in cells after treatment with RuIP at indicated concentrations. (c) Levels of intracellular ATP in 4T1 cells under different conditions. (d) Representative phosphorescence images of 4T1 cells stained with oxygen probe under different treatment conditions Scale bar: 20 μm. (e) Immunofluorescence images of 4T1 cells after incubation with various concentrations of RuIP (0–50 μg/mL) and stained by anti-PD-L1 antibody. Scale bar: 20 μm. (f) The level of ROS in 4T1 cells stained with DCFH probe after indicated treatments under hypoxia conditions. Scale bar: 20 μm. Immunofluorescence imaging of 4T1 cells stained with (g) CRT and (h) HMGB1 antibodies after indicated treatments. Scale bars are 20 μm in (f–h). The blue signal in all images corresponds to Hoechst 33342-stained nuclei. ∗∗indicates P < 0.01 and ∗indicates P < 0.05 vs control group.

Previous studies reported higher concentration of H_2_O_2_ in tumor cells than normal cells [49,50]. Therefore, we studied the *in vitro* catalytic decomposition of H_2_O_2_ by RuIP nanohybrids as a potential solution to elevate endogenous O_2_ level and relieve tumor hypoxia. Western blotting analysis was performed to study the expression of HIF-1α, a protein closely related to O_2_ concentration. As shown in Fig. 4a, under hypoxic conditions, the RuIP nanohybrids treated cells showed a concentration-dependent downregulation of HIF-1α expression, implying that RuIP can reverse hypoxic TME to enhance PDT. Furthermore, O_2_ concentration in the cells incubated with the RuIP nanohybrids under hypoxic conditions was examined by using the O_2_-quenching phosphor oxygen probe (Ru). Under hypoxic conditions, the 4T1 cells-treated with RuIP nanohybrids exhibited a weaker red fluorescence than the control group (Fig. 4d), suggesting an improved intracellular O_2_ concentration to relieve tumor hypoxia and facilitate sustained PDT effects. Meanwhile, Fig. 4b indicated concentration-dependent GSH depletion by RuIP as intracellular GSH level was reduced to 28.6 μM in RuIP (50 μg/mL)-treated group than the control group (43 μM). Owing to improved intracellular O_2_ concentration and GSH depletion, RuIP triggered high ROS generation *in vitro* as confirmed by DCFH assay. As demonstrated in Fig. 4f, the highest green fluorescence intensity was observed in the RuIP + L group than the control and IR825+L groups, verifying robust intracellular ROS production *in vitro*.

Because of improved phototherapeutic performance of RuIP nanohybrids, we next determined whether RuIP can trigger ICD. Different markers of ICD such as high mobility group box 1 protein (HMGB1), calreticulin (CRT), and adenosine tRuIPhosphate (ATP) were analyzed by in immunofluorescence staining and ATP detection kit. A significantly higher CRT expression was observed in RuIP + L group than all other groups as shown in Fig. 4g. Whereas both IR825+L and RuIP + L groups showed reduced expression of HMGB1 protein and ATP levels than the control group (Fig. 4h and c), suggesting phototherapy-mediated ICD by RuIP. Since RuIP nanohybrids can alleviate tumor hypoxia through catalytic O_2_ generation, immunofluorescence staining presented a decrease in PD-L1 expression in RuIP-treated group than the control group (Fig. 4e and S9), confirming that RuIP-treated cancer cells are more susceptible to be captured and cleared by cytotoxic T lymphocytes (CTLs) due to the inhibition of the PD-1/PD-L1 axis.

**Fig. 5:**
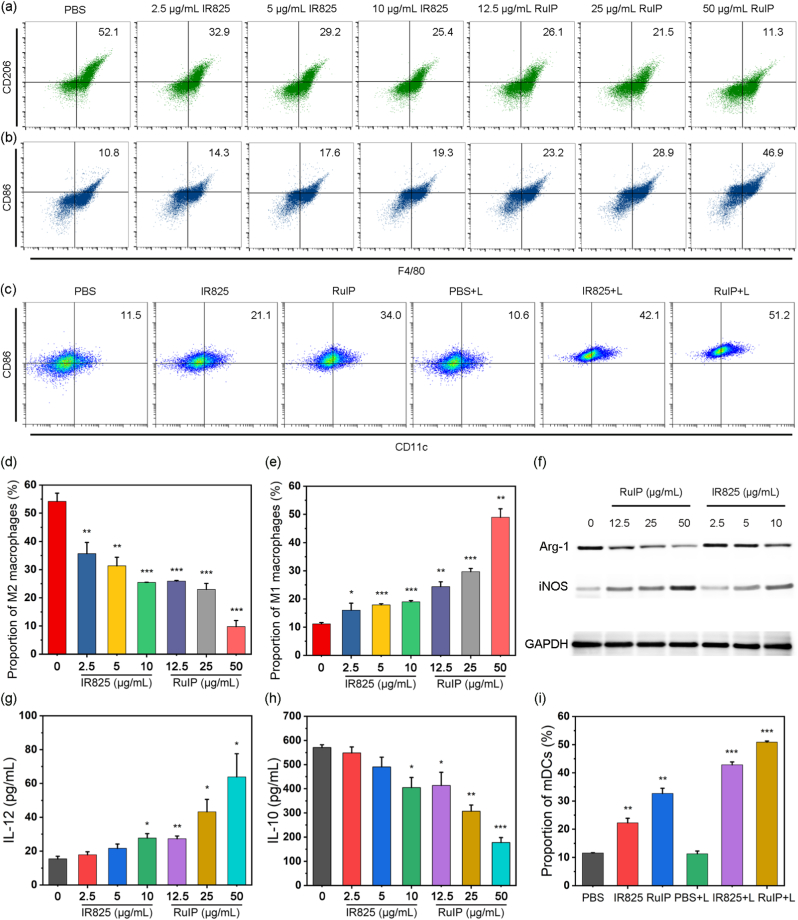
Flow cytometry evaluation of (a) M2 (stained with F4/80 and CD206) and (b) M1 (stained with F4/80 and CD80) macrophages percentage from indicated groups. (c) Flow cytometry analysis of DC maturation after various treatments. Quantitative analysis of (d) M2 and (e) M1 macrophage percentage from indicated groups presented in (a) and (b). (f) Western blot analysis of Arg-1 in IL-4 activated RAW 264.7 cells (M2 macrophages), and iNOS in LPS activated RAW 264.7 cells (M1 macrophages) after treatment with RuIP or IR825 at indicated concentrations. RuIP and IR825 altered the (g) IL-12 and (h) IL-10 secretion of M2 or M1 macrophages as measured by ELISA. (i) Quantitative analysis of DC maturation after various treatments as mentioned in (c). ∗∗∗indicates P < 0.001, ∗∗indicates P < 0.01 and ∗indicates P < 0.05 vs control group.

TAM polarization plays an important role in tumor immunotherapy. The polarization of immunosuppressive M2 macrophages can reduce the efficiency of immunotherapy, and the polarization of immune-activated M1 macrophages can promote immunotherapy. It has been reported that the alleviation of hypoxic TME and elevated ROS level can induce TAMs to transform from M2 to M1 [17]. Therefore, the role of RuIP nanohybrids in reprogramming TAMs from protumorigenic M2 phenotype to antitumor M1 phenotype was evaluated. RAW264.7 cells were incubated with IL-4 or LPS for 24 h to establish M2 or M1 macrophages, respectively. Flow cytometry analysis indicated ∼11.3 % decreased proportion of CD206 (M2 macrophage marker, Fig. 5a and d) and ∼46.9 % increased proportion of CD86 (M1 macrophage marker, Fig. 5b and e) in RuIP-treated cells than IR825 (25.4 % and 19.3 %), suggesting that RuIP nanohybrids can reprogram TAMs from M2 to antitumor M1. Subsequently, the expression of typical markers iNOS (M1 TAMs), and Arg1 (M2 TAMs) were examined by using western blot assay. Compared to IR825, a significant increase in iNOS expression was seen after RuIP treatment (Fig. 5f and S10a). Whereas, M2 TAMs marker Arg-1 showed reduced expression after RuIP treatment (Fig. 5f and S10b), verifying the polarization of M2 TAMs to M1 TAMs by RuIP. Meanwhile, inflammatory cytokines such as IL-12 and IL-10 also showed similar up-regulated (Fig. 5g) and down-regulated (Fig. 5h) expression after treated with different concentrations of RuIP nanohybrids. In addition, the M0 macrophages showed significantly higher levels of intracellular ROS after incubation with RuIP compared to the control cells and the cells treated with IR825 (Fig. S11). Besides, the proportion of mature dendritic cells (mDCs) was significantly higher in both IR825+L (42.3 %) and RuIP + L-treated groups (50.9 %) than the control (11.6 %) group, in which the later showed the superior maturation of DCs (Fig. 5c and i). These results implied that RuIP can induce ICD through improved phototherapy, reprogram TAM to reverse immune suppression, and present tumor antigens to DC to strengthen antitumor immunity.

**Fig. 6:**
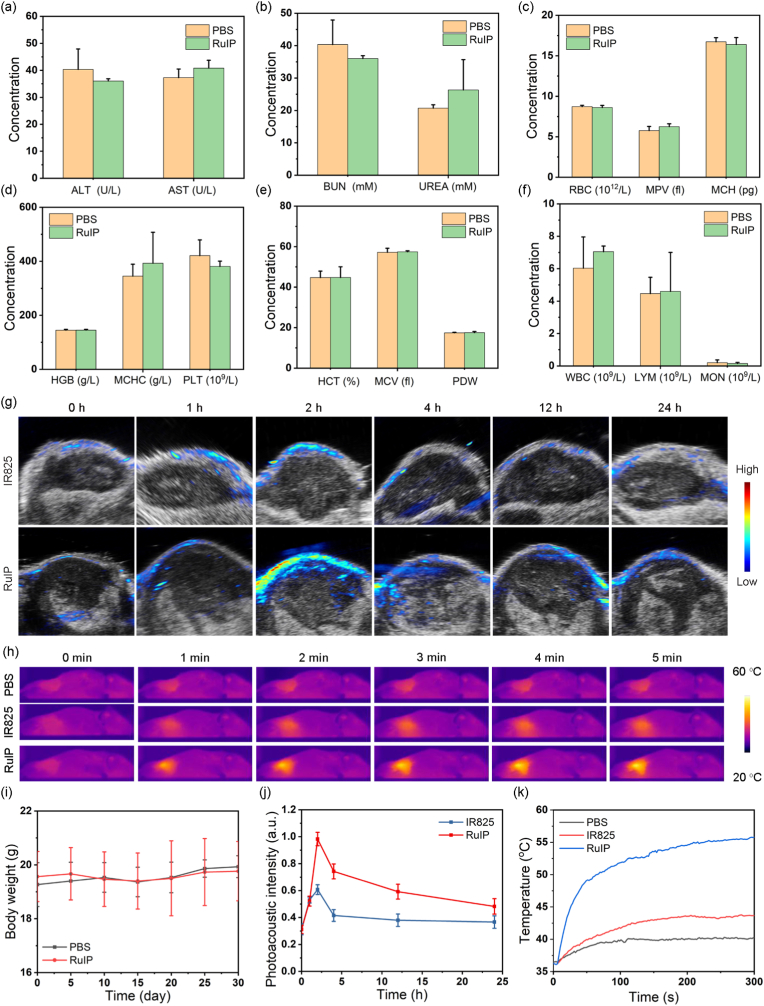
The level of liver (a) and kidney (b) markers in mice after indicated treatments. (c–f) The levels of hematological parameters after indicated treatments. (g) Photoacoustic imaging and (j) the quantitative analysis of photoacoustic images after intravenous (*i.v.*) injection of RuIP or free IR825 in 4T1 tumor-bearing mice. (h) Photothermal imaging and (k) quantitative analysis of localized hyperthermia in indicated groups after NIR laser excitation for 5 min. (i) Change of body weight in PBS- or RuIP- treated mice during 30 days.

The promising *in vitro* phototherapeutic performance of RuIP nanohybrids encouraged us to explore their *in vivo* potential to treat hypoxic tumor. Therefore, we studied the hemolytic behavior and *in vivo* biocompatibility of RuIP to determine whether it's safe for applying in biological system. As shown in Fig. S12, RuIP even at a higher concentration of 4 mg/mL caused less than 5 % damage to red blood cells as determined by hemolysis assay [51]. For *in vivo* biocompatibility, healthy mice were intravenously administered with PBS and RuIP and the changes in the body weight were recorded till one month. Similar to PBS group, RuIP-treated mice did not show any decrease in the body weight even after 30 days (Fig. 6i), indicating good *in vivo* biocompatibility of RuIP. After 30 days, all mice were sacrificed and the whole blood and major organs were collected for hematological and histopathological analyses. All hematological parameters and liver/kidney markers were in the normal range in both PBS and RuIP groups (Fig. 6a–f). Similarly, histopathological staining did not show any sign of systemic toxicity to the major organs after RuIP treatment (Fig. S13). Moreover, within 24 and 48 h, the feces and urine of the mice were collected. The results showed that the ruthenium element excluded from the body also increased over time. The ruthenium content in the feces and urine of the mice injected for 48 h reached 50.4 % and 13.3 % of the injection dose respectively (Fig. S14), indicating that these nanohybrids can be excreted from the body through the kidneys, especially the liver. These findings confirmed that RuIP did not elicit adverse effects to the major organs of mice and is safe for *in vivo* therapeutic applications. Therefore, we established 4T1 tumor-bearing mice model and first studied the tumor enrichment of RuIP nanohybrids using photoacoustic imaging. Fig. 6g indicated time-dependent enhancement of photoacoustic signals by RuIP under laser excitation. The maximum photoacoustic signal was recorded at 2 h post-injection of IR825 and RuIP, which was gradually decreased with time (Fig. 6j). However, due to poor water solubility and tumor targeting, IR825 showed ∼1.6 fold less tumor enrichment than RuIP as recorded by photoacoustic imaging. Next, we performed *in vivo* pharmacokinetic profiles and biodistribution study by collecting blood, the major organs, and tumor tissues from 4T1 tumor-bearing mice. The first and second phase blood circulation half-lives for RuIP were determined to be 0.73 ± 0.05 and 8.3 ± 0.5 h, respectively (Fig. S15). As shown in Fig. S16, ICP-MS analysis showed that 1.8 % ID/g of RuIP were accumulated in the tumor tissue at 24 h post-injection. Meanwhile, after NIR laser excitation for 5 min, these tumor-enriched RuIP triggered localized hyperthermia (55.7 °C) in 4T1 tumor-bearing mice than PBS (40.3 °C) and free IR825 (43.7 °C) groups (Fig. 6h and k) as recorded by photothermal imaging.

**Fig. 7:**
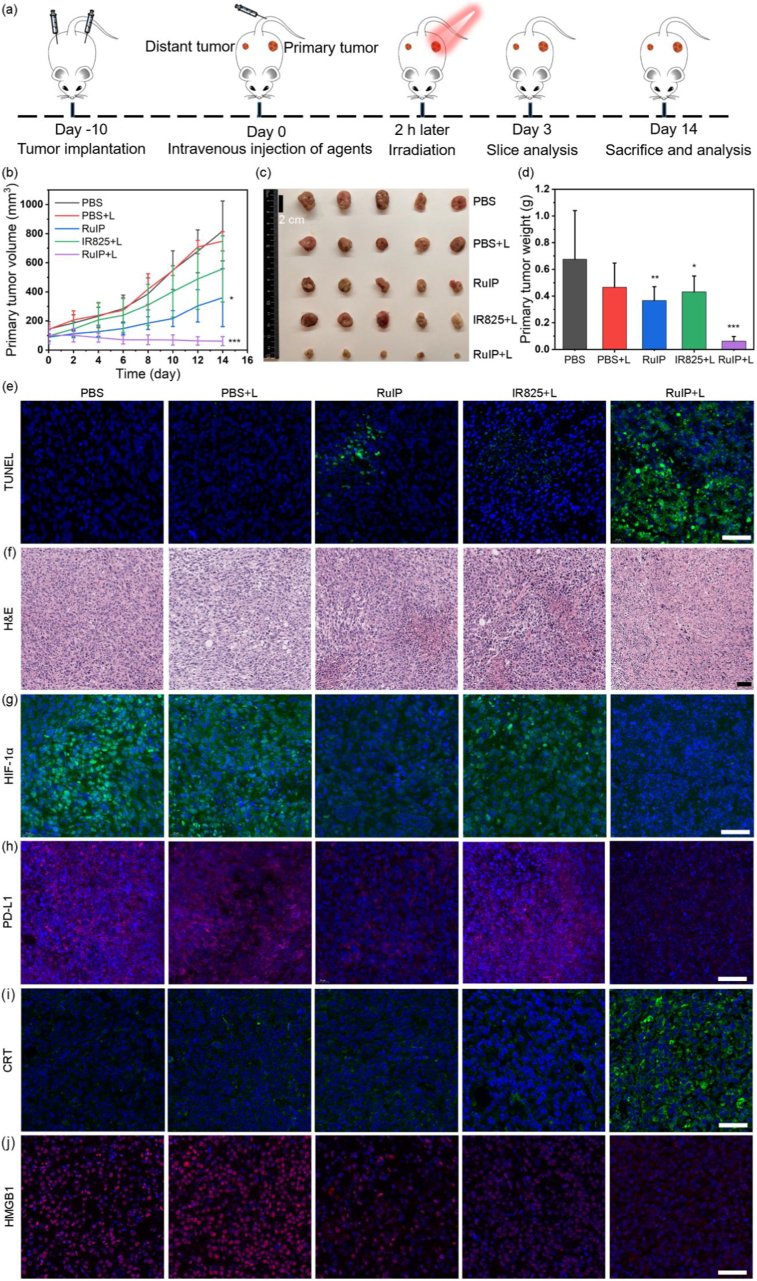
(a) Schematic illustration of RuIP-based photo/immunotherapy. (b) Relative tumor volume (c) digital photographs, and (d) weight of primary tumors after various treatments (n = 5). (e) TUNEL staining of primary tumors after different treatments. (f) Representative H&E-stained images of primary tumors after different treatments. (g) Immunofluorescence staining of primary tumor tissues stained with HIF-1α antibody after indicated treatments. Representative immunofluorescence images of primary tumor tissues stained with (h) PD-L1, (i) CRT or (j) HMGB1 antibody. Scale bars are 50 μm in (e–g) and 100 μm in (h–j). ∗∗∗indicates P < 0.001, ∗∗indicates P < 0.01 and ∗indicates P < 0.05 vs control group.

Considering the good tumor enrichment and localized photothermal effect, *in vivo* photo-immuntherapeutic effects of RuIP nanohybrids were studied using bilateral tumor-bearing mice, the therapeutic diagram as presented in Fig. 7a. All mice were divided into five groups after intravenous injection of the corresponding treatment agents (PBS, PBS + L, RuIP, IR825+L, and RuIP + L). Among them, PBS + L, IR825+L, and RuIP + L groups were exposed to NIR laser excitation for 10 min at 2 h post-injection. The antitumor therapeutic effects were evaluated by recording the tumor volumes of all mice every two days. The control groups exhibited rapid increase in tumor volume (Fig. 7b), while a partial inhibition in tumor growth was seen in IR825+L group. In contrast, RuIP + L significantly inhibited the growth of primary tumor after 2-weeks as confirmed by the digital photographs and tumor weight analysis (Fig. 7c–d). The tumor sections harvested from different groups after 3-days were underwent further staining analysis. TdT-mediated dUTP nick-end labeling (TUNEL) staining displayed significant number of apoptotic cells in RuIP + L group as compared to fewer and negligible apoptosis in IR825+L group and control groups as determined by the green fluorescence (Fig. 7e and S17a). In addition, H&E staining further endorsed the TUNEL staining. Compared to the control groups and IR825+L, an obvious cytoplasmic damage and nucleus shrinkage were observed in the specimens collected from RuIP + L group (Fig. 7f and S17b), suggesting superior phototherapeutic effects of Ru-coordinated photodynamic drug than free IR825.

**Fig. 8:**
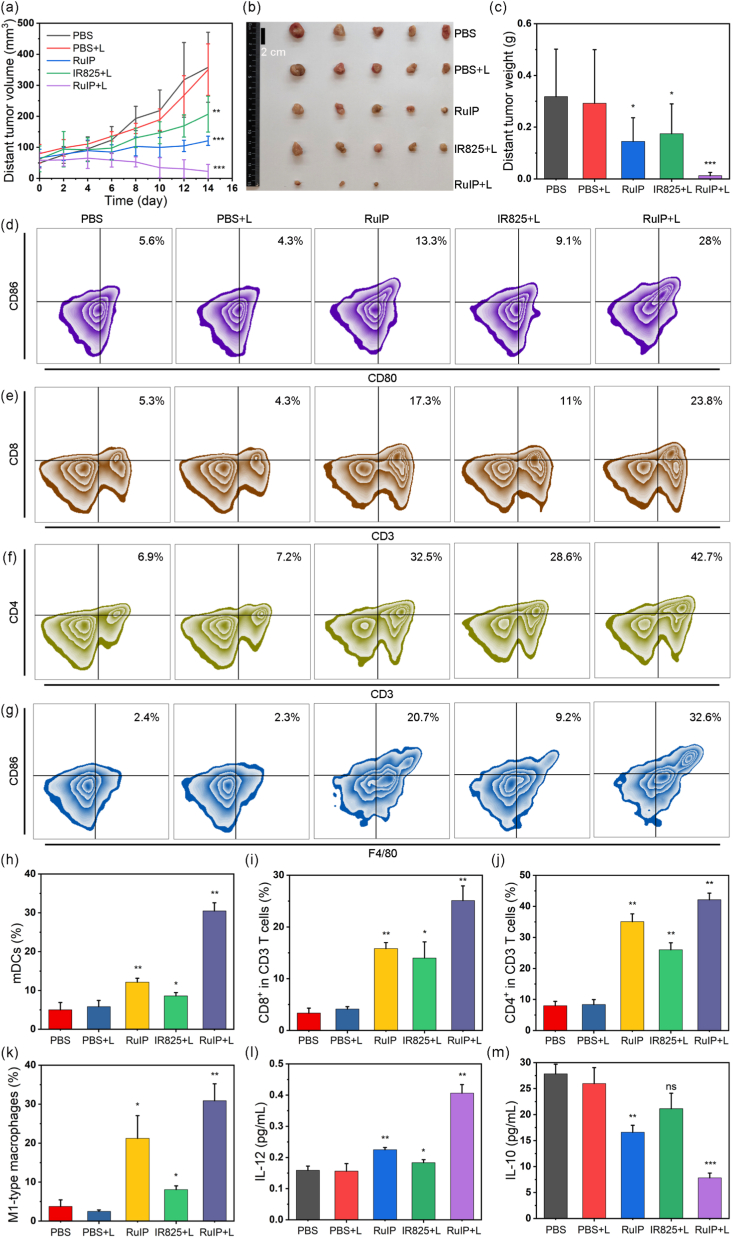
(a) Tumor volumes (b) digital photograph, and (c) tumor weight analyses of distant tumors collected from various treatment groups (n = 5). (d) Frequency of mDCs in indicated groups as measured by flow cytometry (n = 3). Frequencies of (e) T cells (CD3^+^, CD8^+^), (f) T cells (CD3^+^, CD4^+^), and (g) M1 macrophages (CD206^+^, CD11b^+^, F4/80^+^) after different treatments (n = 3). (h–k) Quantitative analysis of cell frequencies in (d–g). The levels of (l) IL-12 and (m) IL-10 in serum collected from different treatment groups (n = 3). ∗∗∗indicates P < 0.001, ∗∗indicates P < 0.01, ∗indicates P < 0.05 and ^ns^indicates P > 0.05 vs control group.

Since hypoxic TME decreases the efficacy of phototherapy and promotes immune suppression by proliferating M2 macrophages, inhibiting mDCs, and impairing the function of T cells, previous studies reported that modulating hypoxic TME can effectively reverse tumor immunosuppression and boost immunotherapy [12]. Hence, HIF-1α immunofluorescence staining was done to study whether RuIP nanohybrids can modulate hypoxic TME *in vivo*. Compared to other groups, a much weaker green fluorescence was seen in both RuIP and RuIP + L groups, suggesting reduced expression of HIF-1α (Fig. 7g and S17c). Such a down-regulation in HIF-1α expression confirmed that RuIP can alleviate tumor hypoxia, which is attributed to the catalytic O_2_ generation ability of RuIP nanohybrids. Concomitantly, HIF-1α regulated the expression of PD-L1 [15,16], and therefore, we observed a decrease in PD-L1 expression in RuIP-treated group as presented in Fig. 7h and S17d, resulting in the activation of CTLs to facilitate immunotherapy. Therefore, we determined phototherapy-induced ICD effects by assessing the expression of CRT and HMGB1, respectively. Compared to other groups, higher expression of CRT and lower expression of HMGB1 were recorded in tumor specimens collected from RuIP-treated group (Fig. 7i–j, and **S17e-f**), suggesting that RuIP nanohybrids elicit phototherapy-mediated ICD through the release of danger associated molecular patterns. Such a strong ICD effect activates the immune system that further suppresses the growth of distant tumor in 4T1-tumor bearing mice as confirmed by the tumor volume, digital photographs, and tumor weight analysis of distant tumor (Fig. 8a–c). Whereas, a slight inhibition of distant tumor was seen in IR825+L and RuIP groups as compared to the control group.

Inspired by the immune activation through phototherapy-mediated ICD effect, immune characterization was performed by first assessing DCs, which play an important role in antitumor immunity by initiating and directing immune responses. The proportion of mDCs (CD11c^+^, CD86^+^, CD80^+^) inside the spleen was characterized by using flow cytometry. Quantitative analysis suggested that RuIP + L treatment promoted 6-fold DCs maturation than the PBS treatment (Fig. 8d and h), whereas RuIP and IR825+L treatment promoted only 2- and 1.4- fold DCs maturation, respectively. Besides, the frequency of cytotoxic CD8^+^ T lymphocytes was significantly increased after treatment with RuIP+L (25.1 %) as compared to other groups, PBS (3.4 %), PBS + L (4.1 %), RuIP (15.8 %), and IR825+L (14 %, Fig. 8e and i). In addition, 5.3-fold higher proportion of helper CD4^+^ T lymphocytes was also recorded in RuIP+L group than PBS group (Fig. 8f and j). Moreover, compared to the control (3.8 %, 10.2 %) and free IR825+L (8 %, 6.7 %) groups,Fig. 8g and k, S18, and S19 displayed higher amount (30.9 %) of immunostimulatory M1 macrophages (CD80^+^, CD11b^+^, F4/80^+^) and lower amount (2.8 %) of immunosuppressive M2 macrophages (CD206^+^, CD11b^+^, F4/80^+^) in RuIP + L-treated group. Meanwhile, ELISA analysis of immunostimulatory (IL-12) and immunosuppressive (IL-10) cytokines further confirmed that RuIP nanohybrids reprograms TAM and reverses immune suppression as shown in Fig. 8l–m. These findings dictated that RuIP nanohybrids are capable to trigger phototherapy-induced ICD and activate antitumor immunity by modulating immunosuppressive TME through alleviating tumor hypoxia.

## 4 Conclusion

In summary, we designed a ruthenium coordinated nanohybrid assembled from ruthenium ions, IR825 and PVP that exhibits synergistic and effective photo- and immuno-therapy via TME modulation. The self-assembled RuIP nanohybrids had high drug loading efficiency, improved the poor water solubility of IR825, and enhanced the photothermal performance of IR825. Due to the catalytic properties of RuIP nanohybrid, it can improve the hypoxic TME by decomposing the excess H_2_O_2_ in the tumor to produce oxygen. At the same time, the nanohybrid can deplete intracellular reductive GSH and generate more ROS under irradiation, which further dramatically improve the efficiency of PDT on hypoxic tumors. Tumor growth and metastasis were significantly inhibited by phototherapy, and modulation of hypoxic TME. More importantly, phototherapy-induced ICD and reversed hypoxia-induced immunosuppressive TME of RuIP nanohybrids allow activate stronger immune responses compared to free IR825, re-programming immunosuppressive, pro-tumoral M2 macrophages to immune-activated, antitumor M1 macrophages, downregulates PD-L1 expression, recruit a large number of immune cells, leading to significant metastatic tumor inhibition. The up-scaling and the long-term toxicity of RuIP nanohybrid materials are the key challenges in their clinical applicability. Nevertheless, the rational design of RuIP nanohybrid present a new avenue to design similar multifunctional nanosystems for collaborative tumor therapies, and is expected to become a promising approach to treat hypoxic tumors.

## CRediT authorship contribution statement

**Jingyao Li:** Writing – original draft, Methodology, Investigation, Formal analysis, Data curation. **Wenzhi Zhu:** Writing – original draft, Validation, Investigation, Formal analysis, Data curation, Conceptualization. **Qibao Zheng:** Writing – original draft, Visualization, Validation, Software, Methodology, Investigation, Data curation, Conceptualization. **Huixi Yi:** Visualization, Validation, Software, Resources, Methodology. **Liyou Guo:** Visualization, Validation, Methodology, Data curation. **Zhixiong Zhan:** Visualization, Software, Resources, Methodology, Investigation, Data curation. **Nannan Fu:** Visualization, Validation, Software, Resources, Methodology, Investigation, Formal analysis. **Muhammad Rizwan Younis:** Writing – review & editing, Supervision, Project administration, Funding acquisition, Conceptualization. **Chengzhi Jin:** Writing – review & editing, Validation, Supervision, Project administration, Funding acquisition, Formal analysis, Conceptualization. **Junqiu Zhai:** Writing – review & editing, Validation, Supervision, Project administration, Funding acquisition, Conceptualization. **Dong-Yang Zhang:** Writing – review & editing, Supervision, Project administration, Funding acquisition, Conceptualization.

## Declaration of competing interest

The authors declare that they have no known competing financial interests or personal relationships that could have appeared to influence the work reported in this paper.

## Data Availability

Data will be made available on request.
