# Supplementary material for: Ruthenium coordinated nanohybrids modulate tumor microenvironment and potentiate amplified phototherapy augmented immunotherapy of hypoxic tumor
Source: Mater Today Bio. 2025 Nov 17;35:102564. doi: 10.1016/j.mtbio.2025.102564 (PMC12671372; doi:10.1016/j.mtbio.2025.102564)
Supplement: Multimedia component 1 [file mmc1.docx]

**Ruthenium coordinated nanohybrids modulate tumor microenvironment and potentiate amplified phototherapy augmented immunotherapy of hypoxic tumor**

Jingyao Li^a,1^, Wenzhi Zhu^a,b,1^, Qibao Zheng^a,1^, Huixi Yi^a^, Liyou Guo^a^, Zhixiong Zhan^a^, Nannan Fu^a^, Muhammad Rizwan Younis^c,*^, Chengzhi Jin^a,*^, Junqiu Zhai^b,*^, Dong-Yang Zhang^a,*^

1. Guangzhou Municipal and Guangdong Provincial Key Laboratory of Molecular Target & Clinical Pharmacology, the NMPA and State Key Laboratory of Respiratory Disease, the Fifth Affiliated Hospital and School of Pharmaceutical Sciences, Guangzhou Medical University, Guangzhou 511436, China
2. School of Pharmaceutical Sciences, Guangzhou University of Chinese Medicine, Guangzhou, 510006, China
3. Department of Chemical and Biomolecular Engineering, University of California - Los Angeles, Los Angeles, California 90095, United States

Corresponding Authors: *ryounis@ucla.edu; chengzhijin@gzhmu.edu.cn; [jqzhai@gzucm.edu.cn](mailto:jqzhai@gzucm.edu.cn); zhangdy7@[gzhmu.edu.cn](mailto:2021991035@gzhmu.edu.cn)

^1^J. Li, W. Zhu, and Q. Zheng. contributed equally to this work.


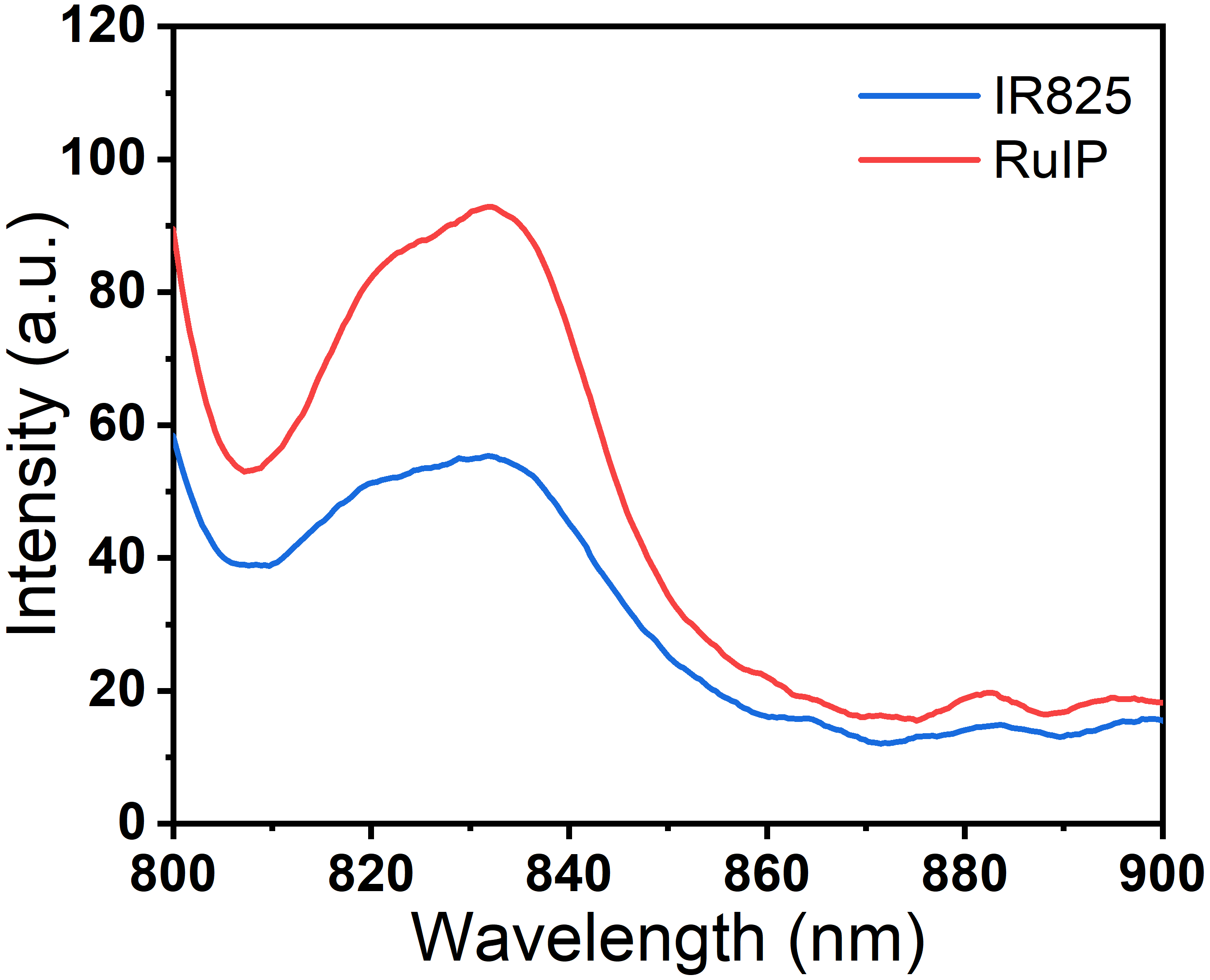


**Figure S1.** Fluorescence spectra of free IR825 and RuIP nanohybrids.


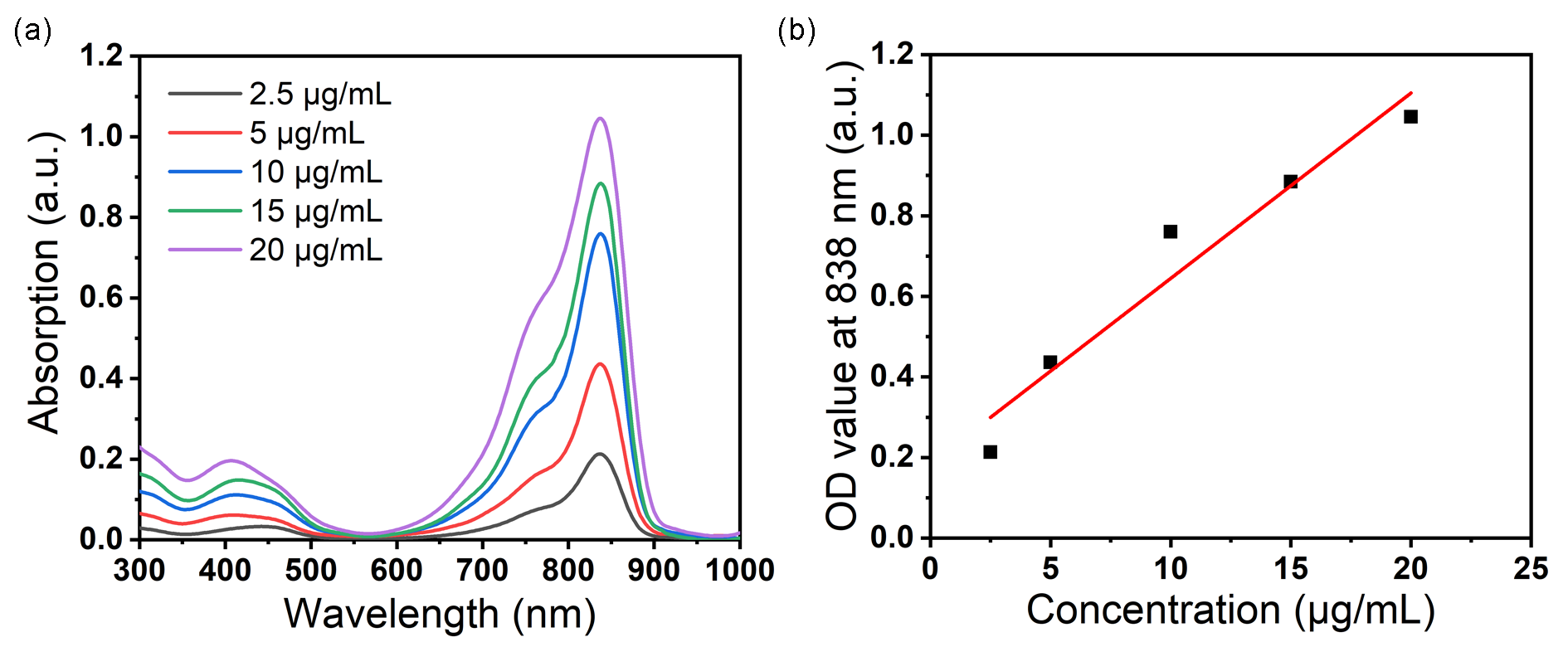


**Figure S2.** (a) Absorption spectra of free IR825 with various concentrations. (b) Standard curve of OD values of IR825 at 838 nm.


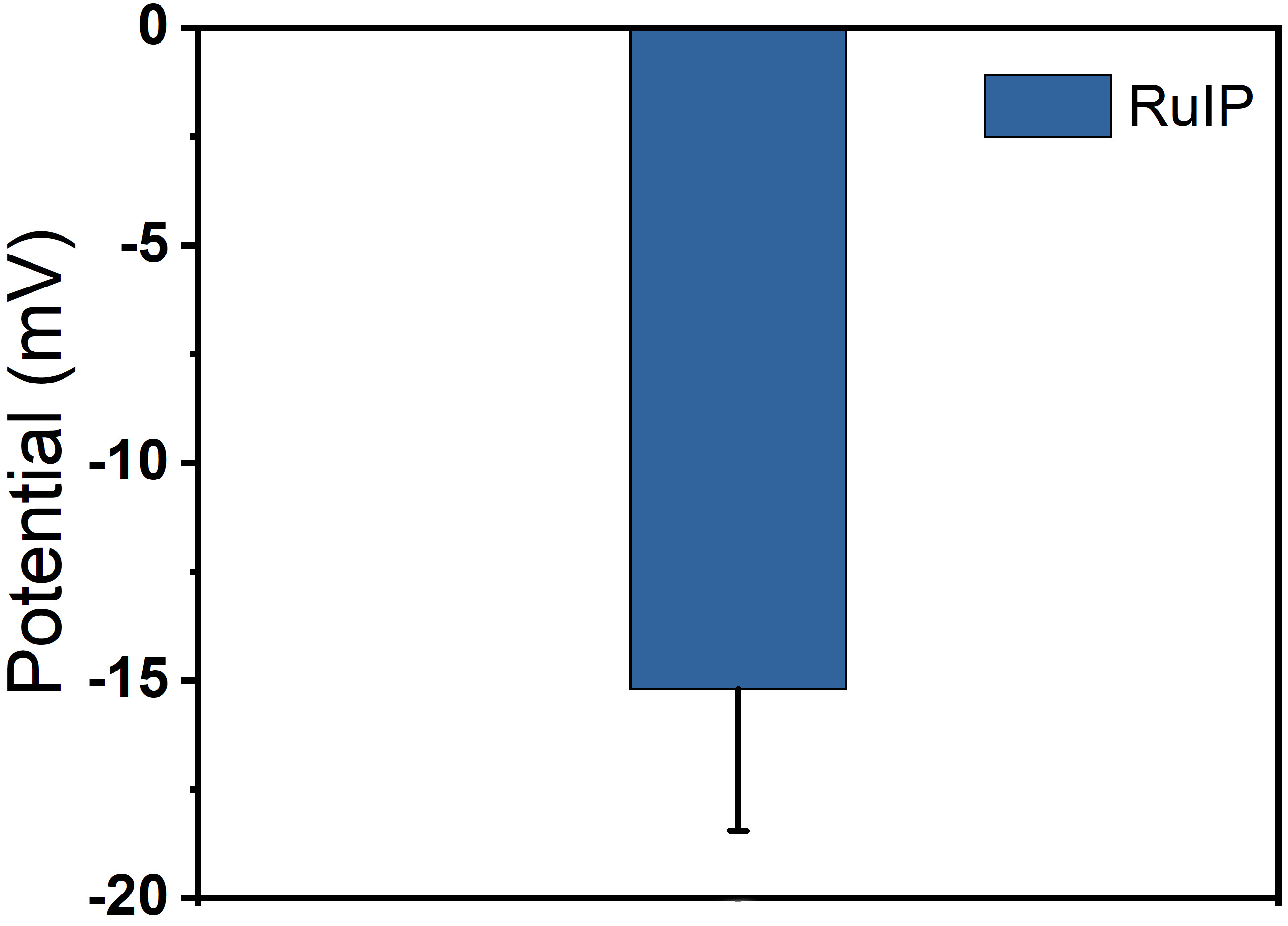


**Figure S3.** Zeta potential of RuIP nanohybrids.


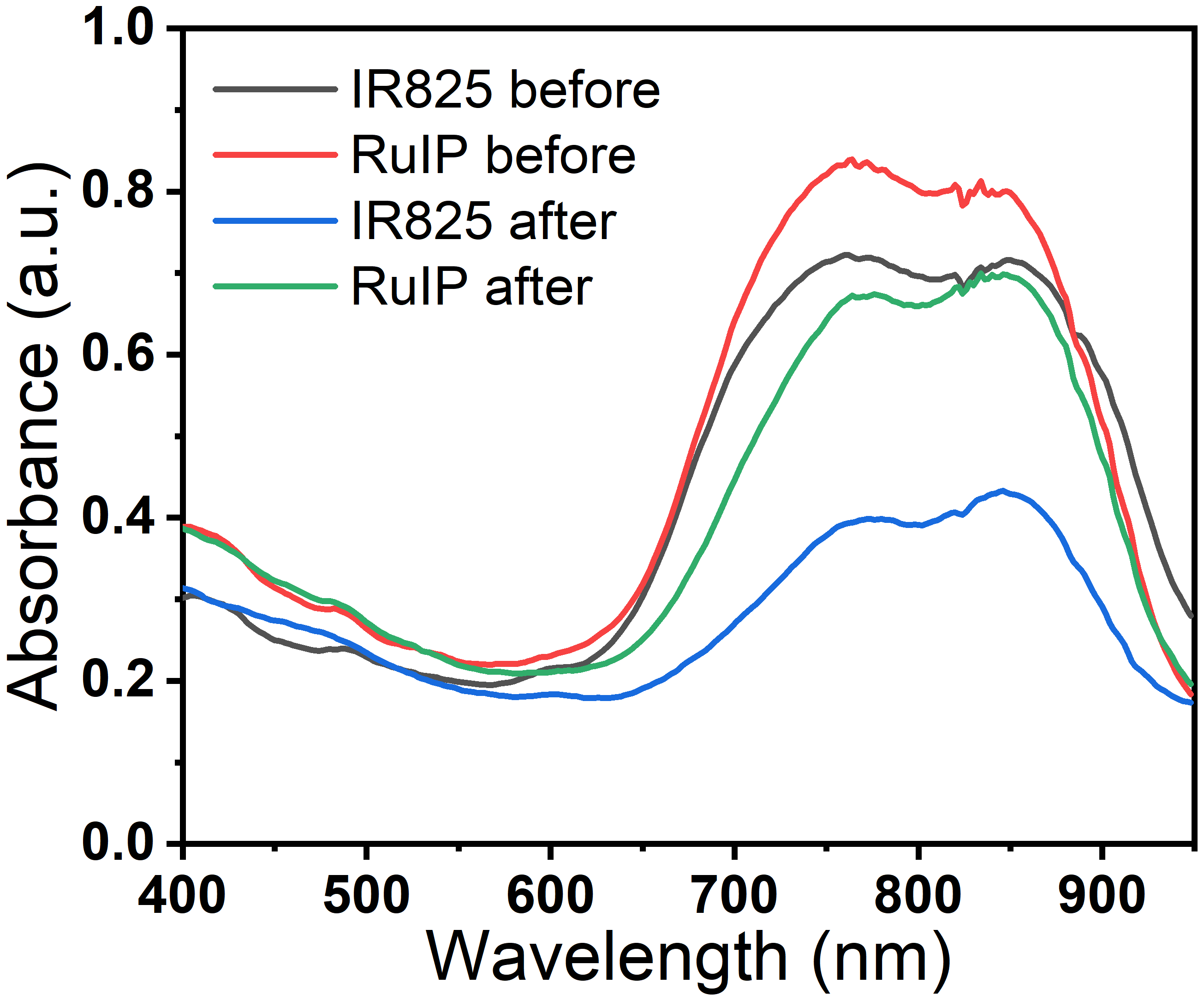


**Figure S4.** Absorption spectra of free IR825 and RuIP before and after irradiation.


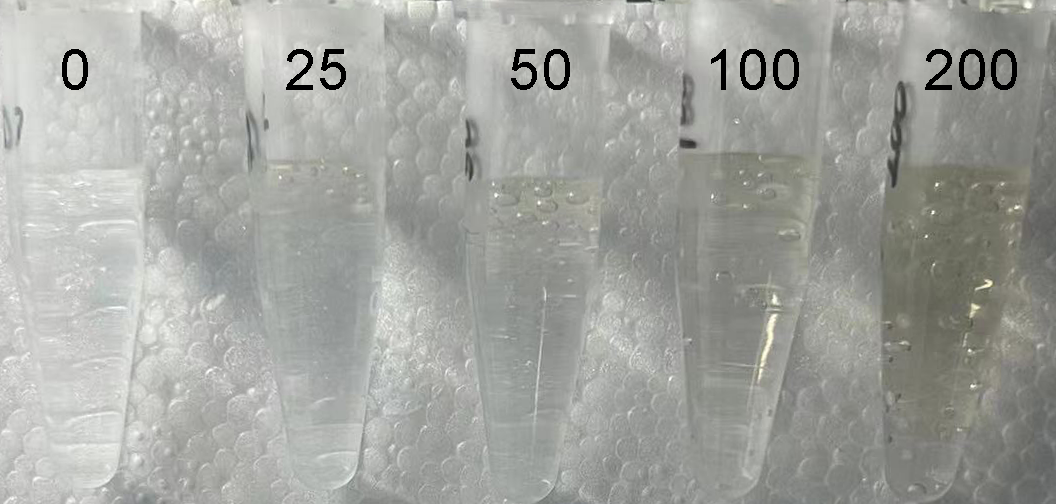


**Figure S5.** Photographs of indicated concentrations of RuIP incubated with H_2_O_2_ (1 M). The unit is μg/mL.


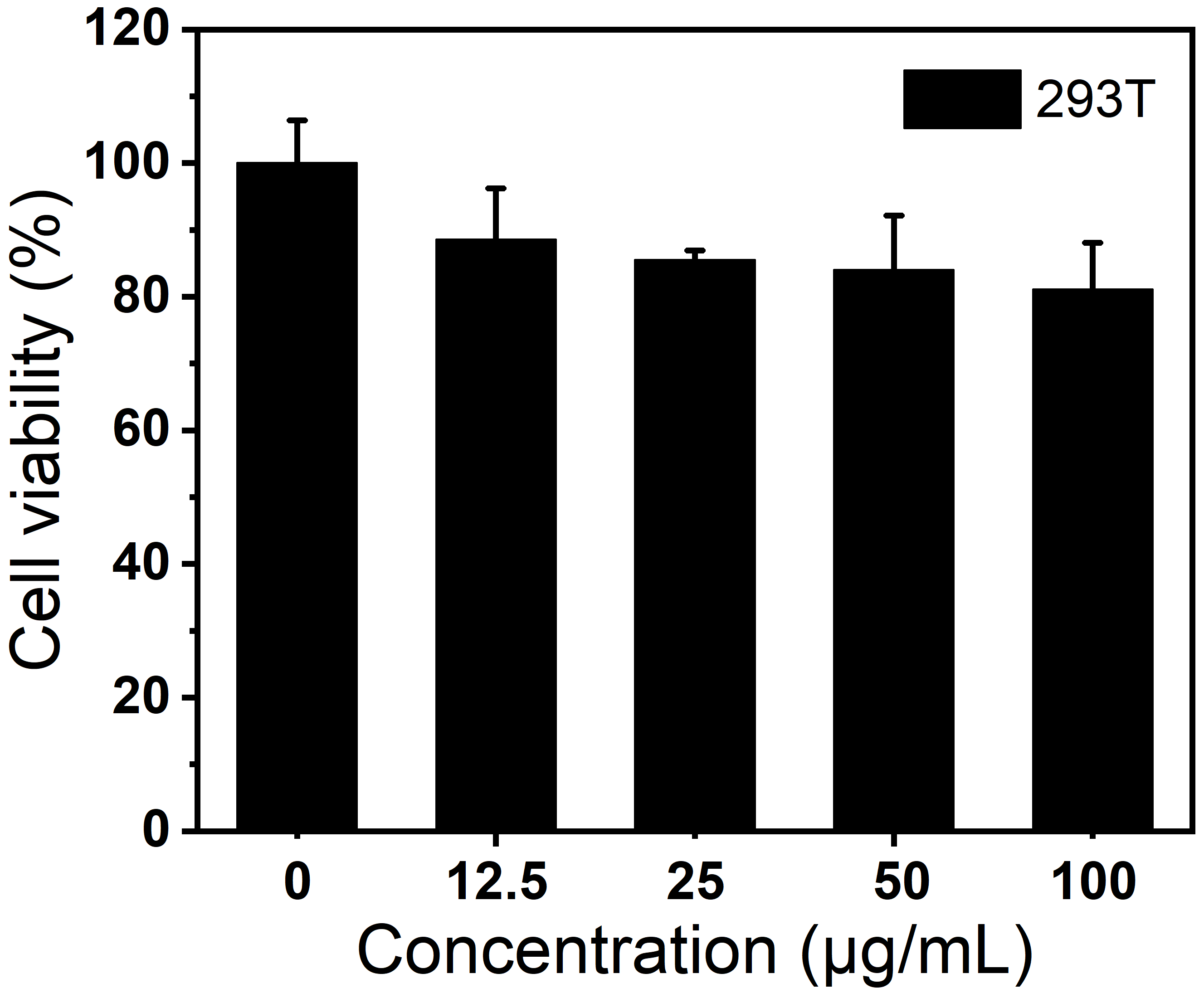


**Figure S6.** The survival rate of HEK293T cells treated with different concentrations of RuIP.


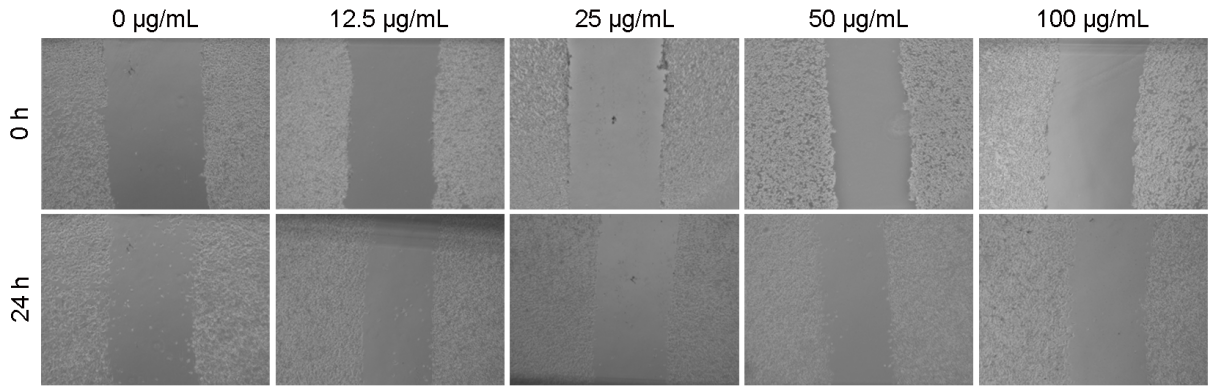


**Figure S7.** 4T1 cells were treated with RuIP at different concentrations for wound healing assay.


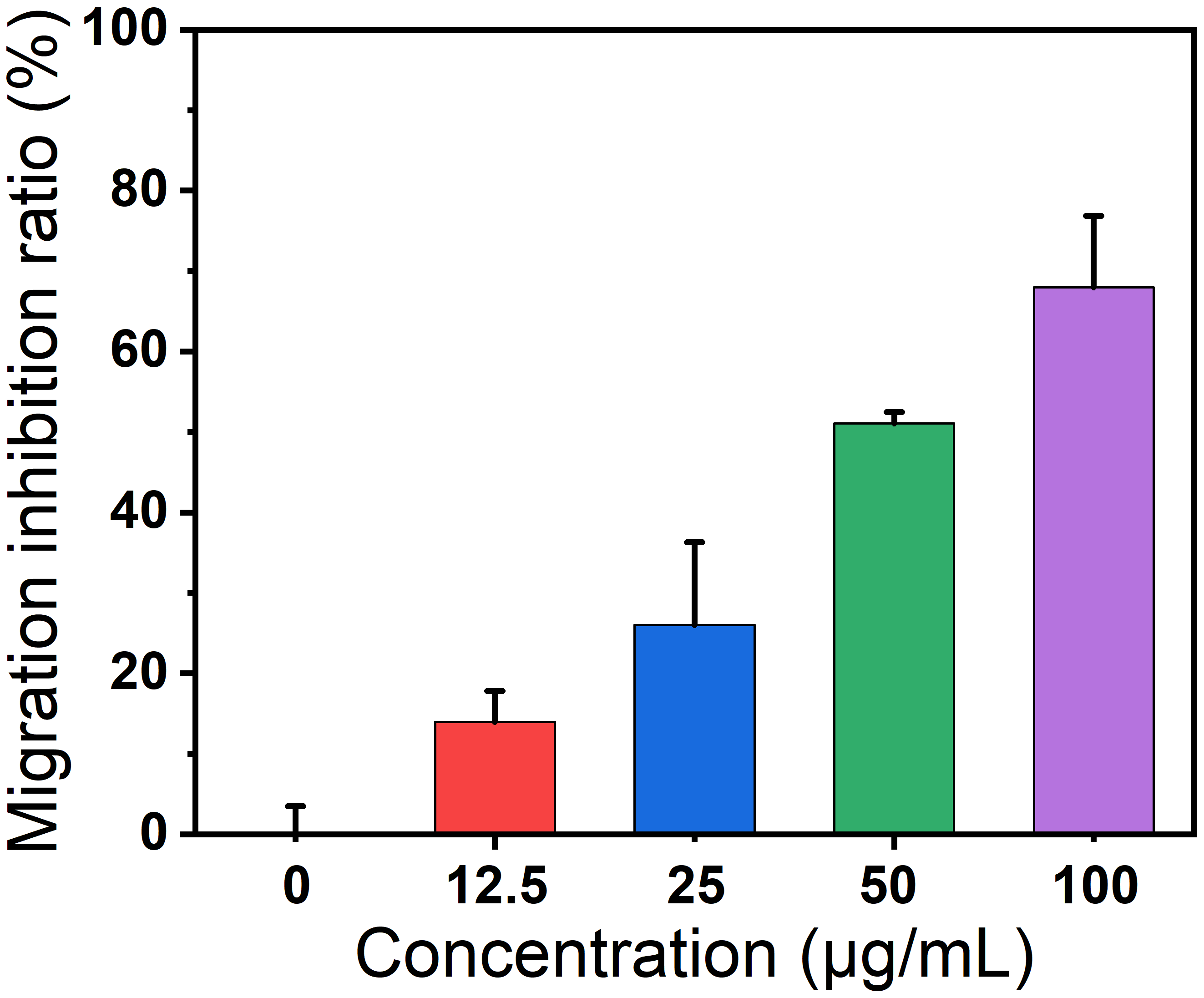


**Figure S8.** Quantitative analysis of wound healing assay after treatment with RuIP.


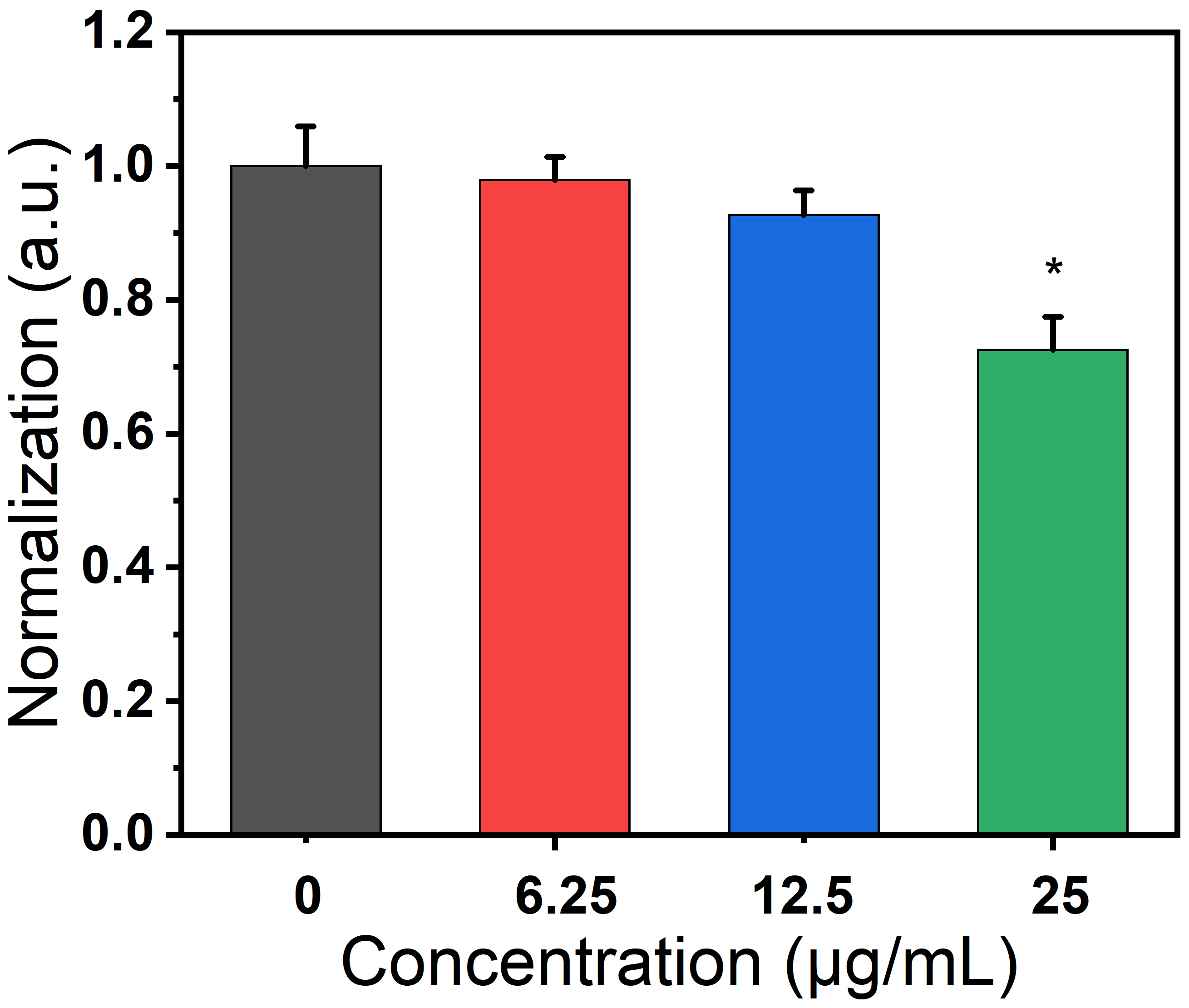


**Figure S9.** Quantitative analysis of PD-L1 fluorescence intensity in 4T1 cells after incubation with various concentrations of RuIP.


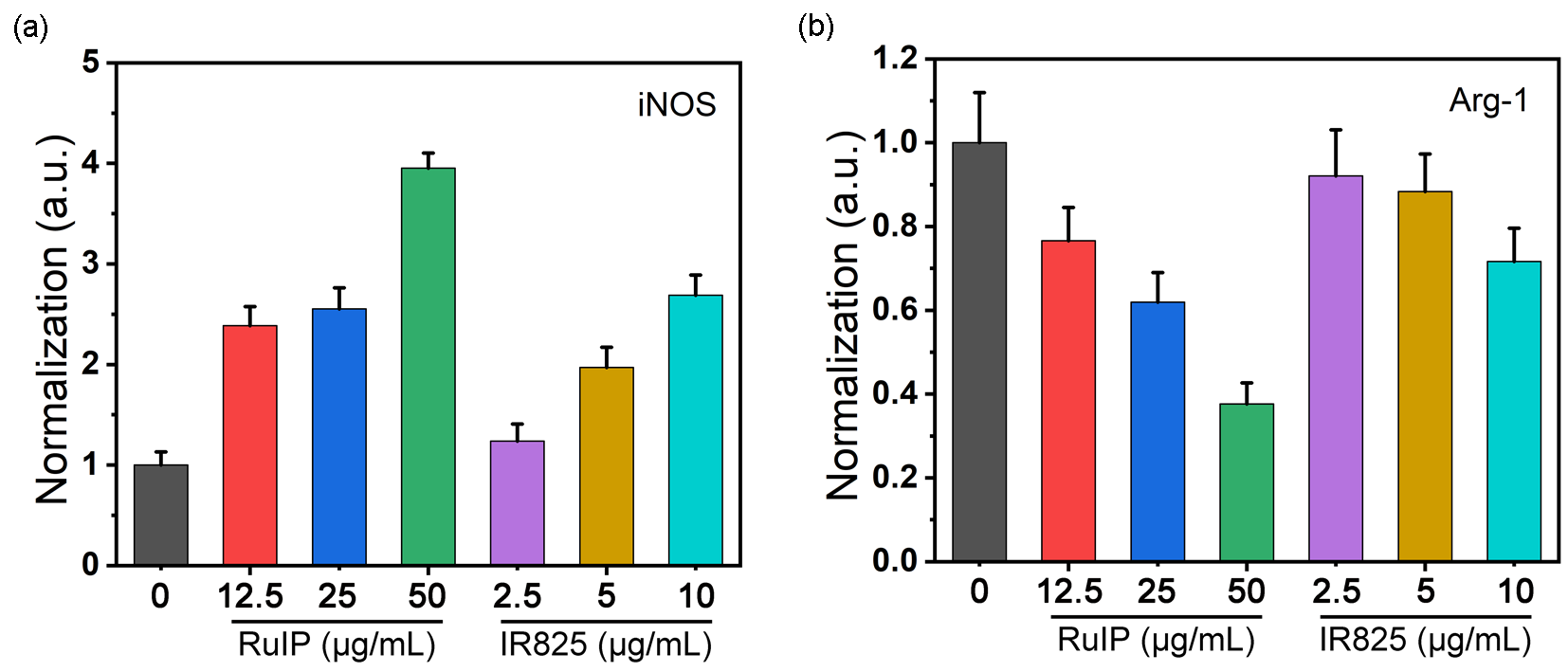


**Figure S10.** Quantitative analysis of the expressions of (a) Arg1 (M2 macrophage) and (b) iNOS (M1 macrophage) after treatment with IR825 or RuIP at indicated concentrations.


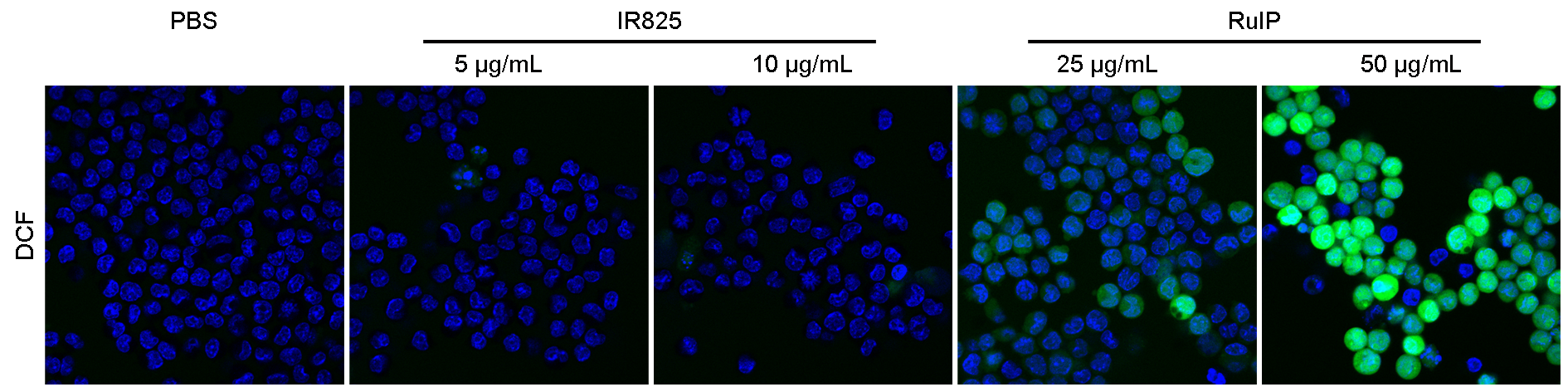


**Figure S11.** The level of ROS in RAW 264.7 cells stained with DCFH probe after indicated treatments. Scale bar: 20 μm.


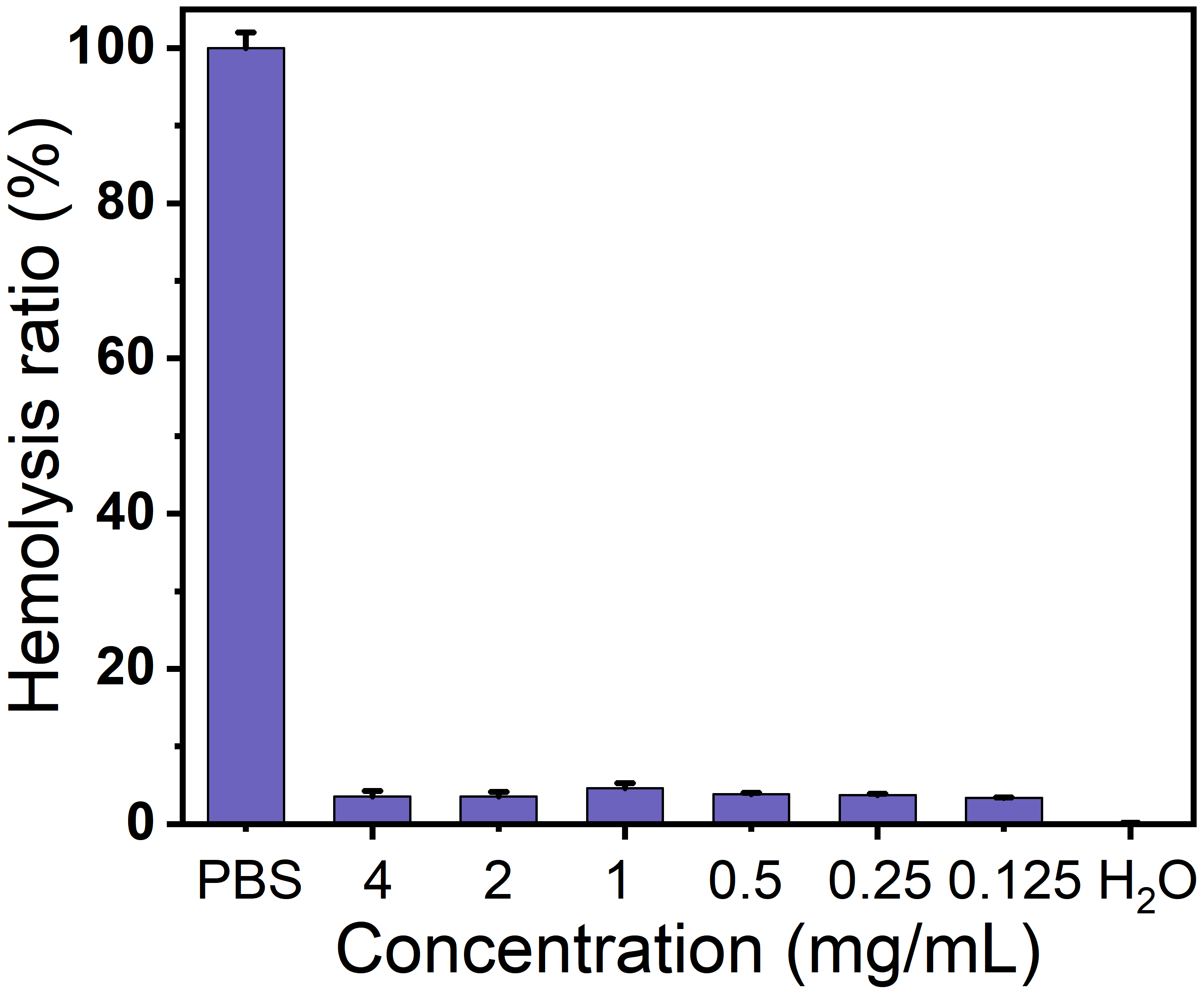


**Figure S12.** Hemolysis ratio of red blood cells after incubation with indicated concentration of RuIP.


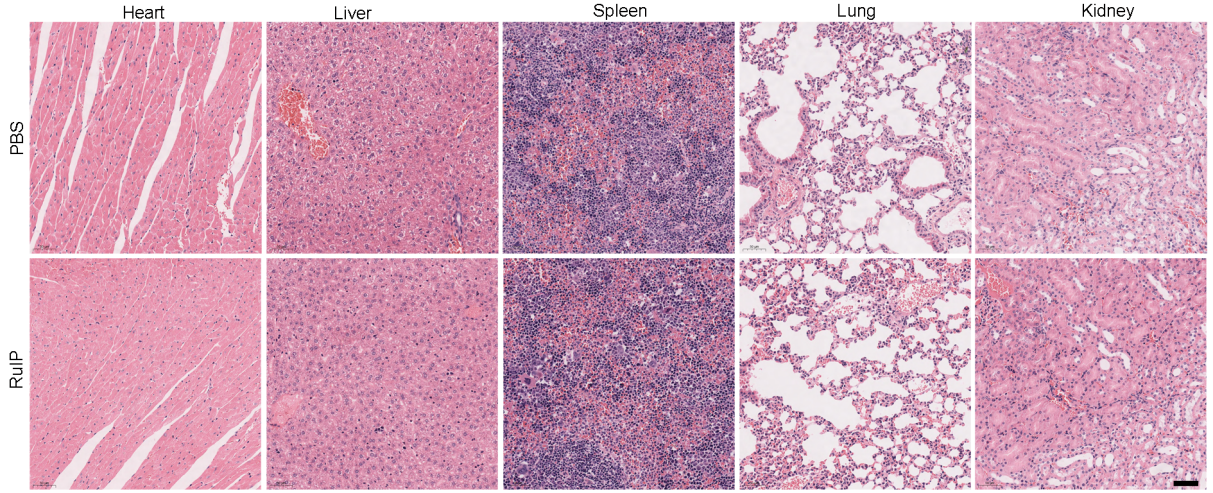


**Figure S13.** H&E stained images of vitals (Heart, Liver, Spleen, Lung and kidney). Scale bar: 50 μm.


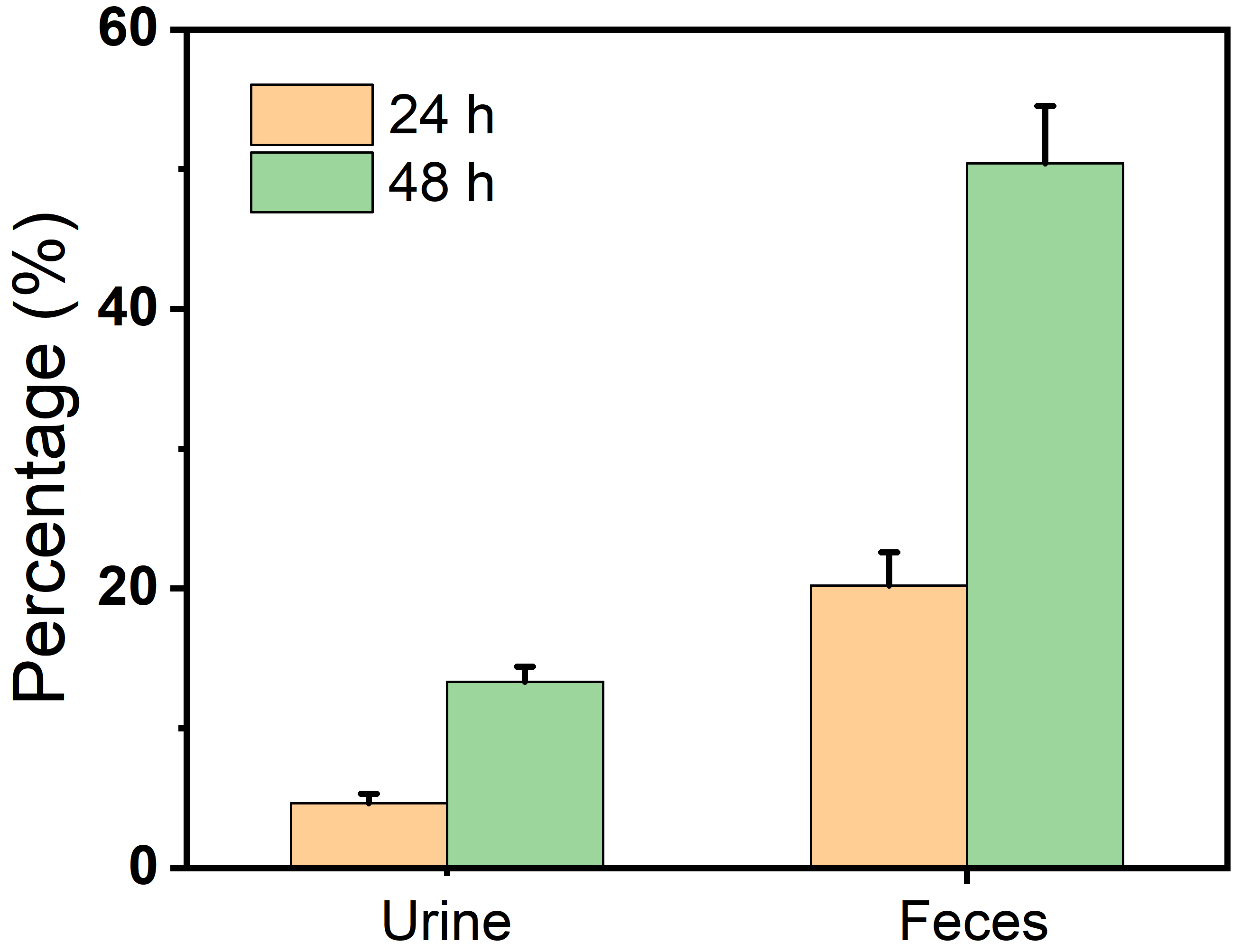


**Figure S14.** The content of ruthenium in urine and feces at indicated time points was measured by ICP-MS.


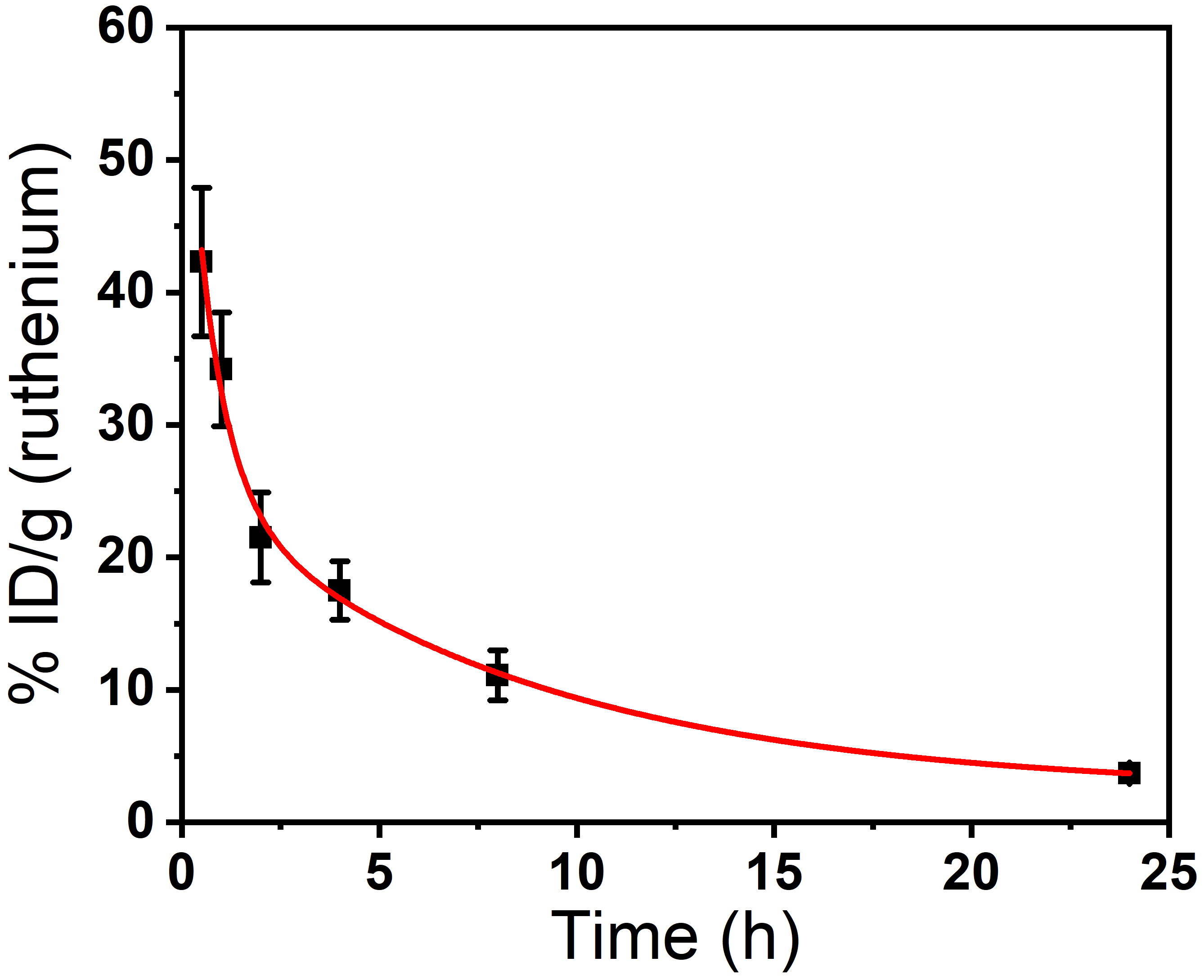


**Figure S15.** The blood circulation of RuIP nanohybrids after intravenous administration (n = 3).


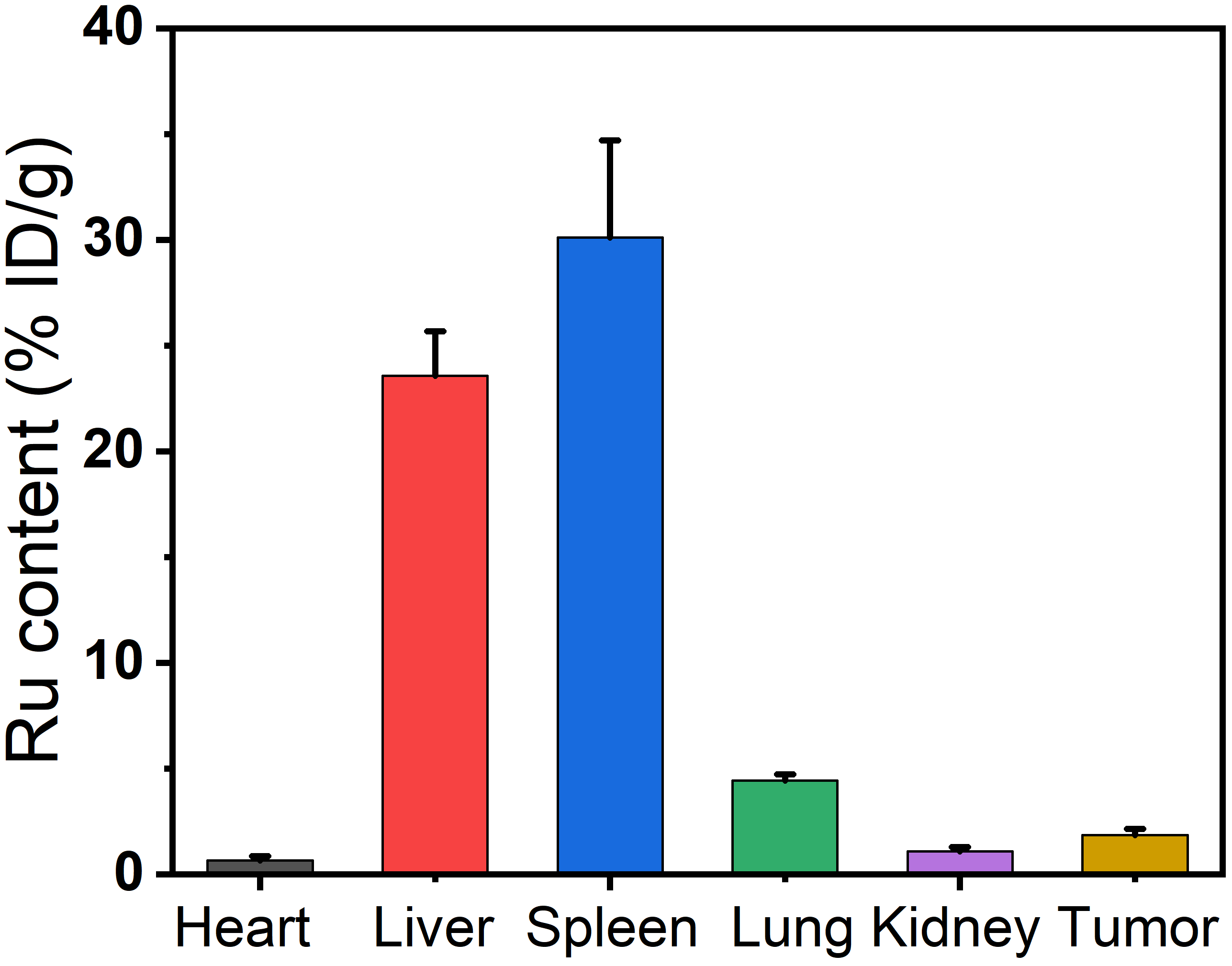


**Figure S16.** ICP-MS analysis of intravenously injected RuIP in 4T1 tumor-bearing mice at different post-injection time points.


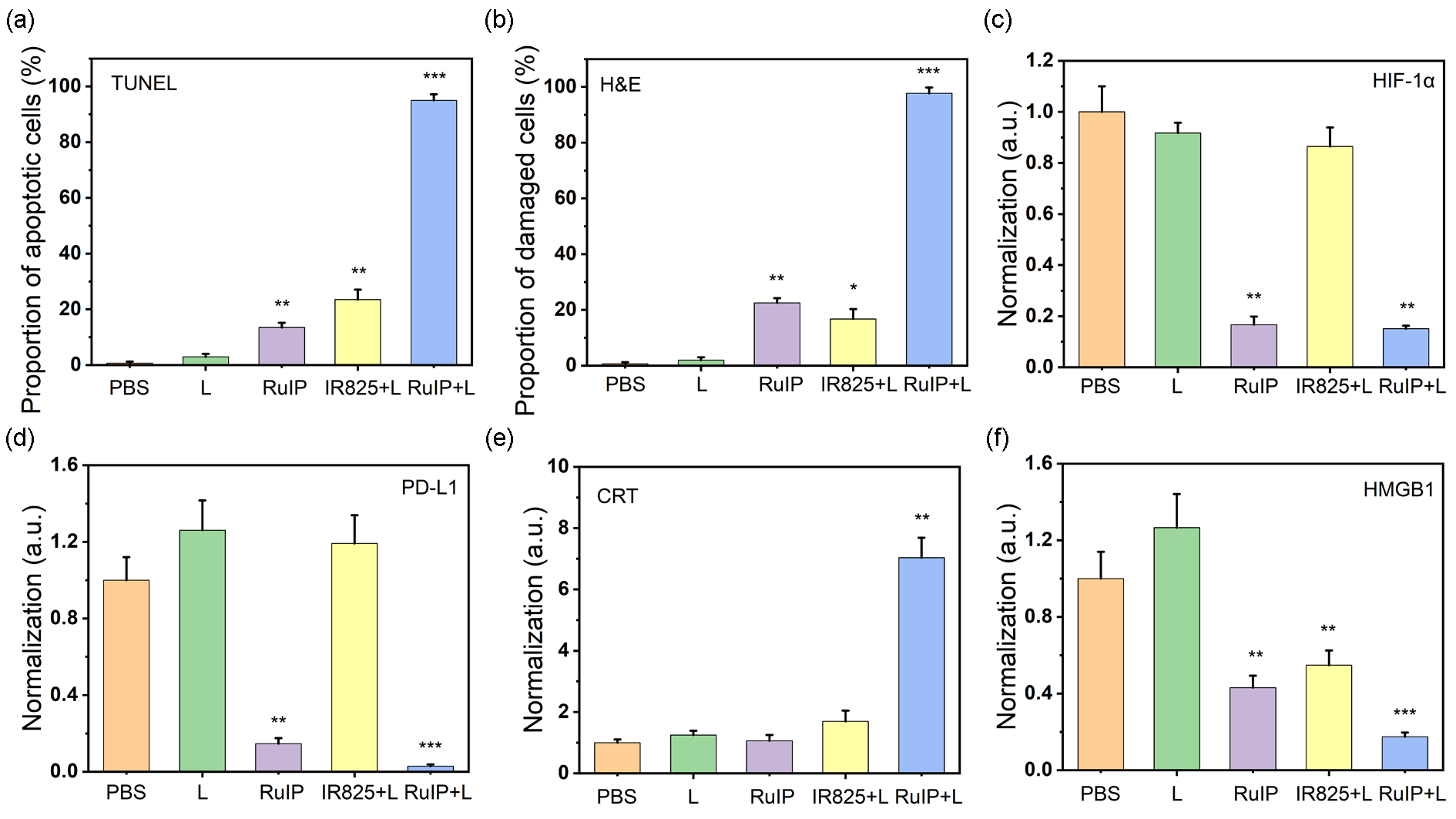


**Figure S17.** Quantitative analysis of the results presented in Figures 7e-7j.


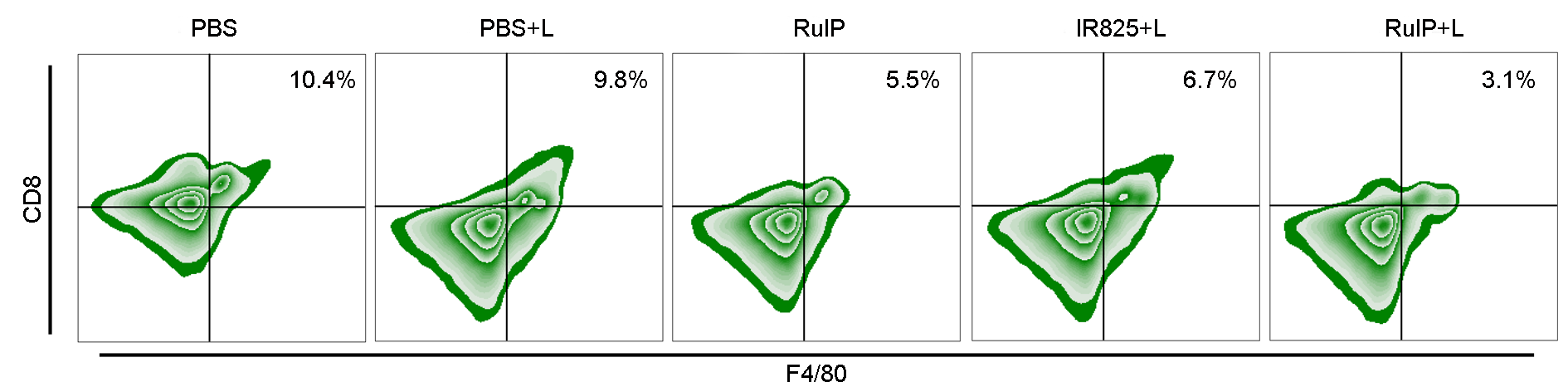


**Figure S18.** Frequency of M2-type macrophages (CD80^+^, CD11b^+^, F4/80^+^) post after different treatments inside the spleens.


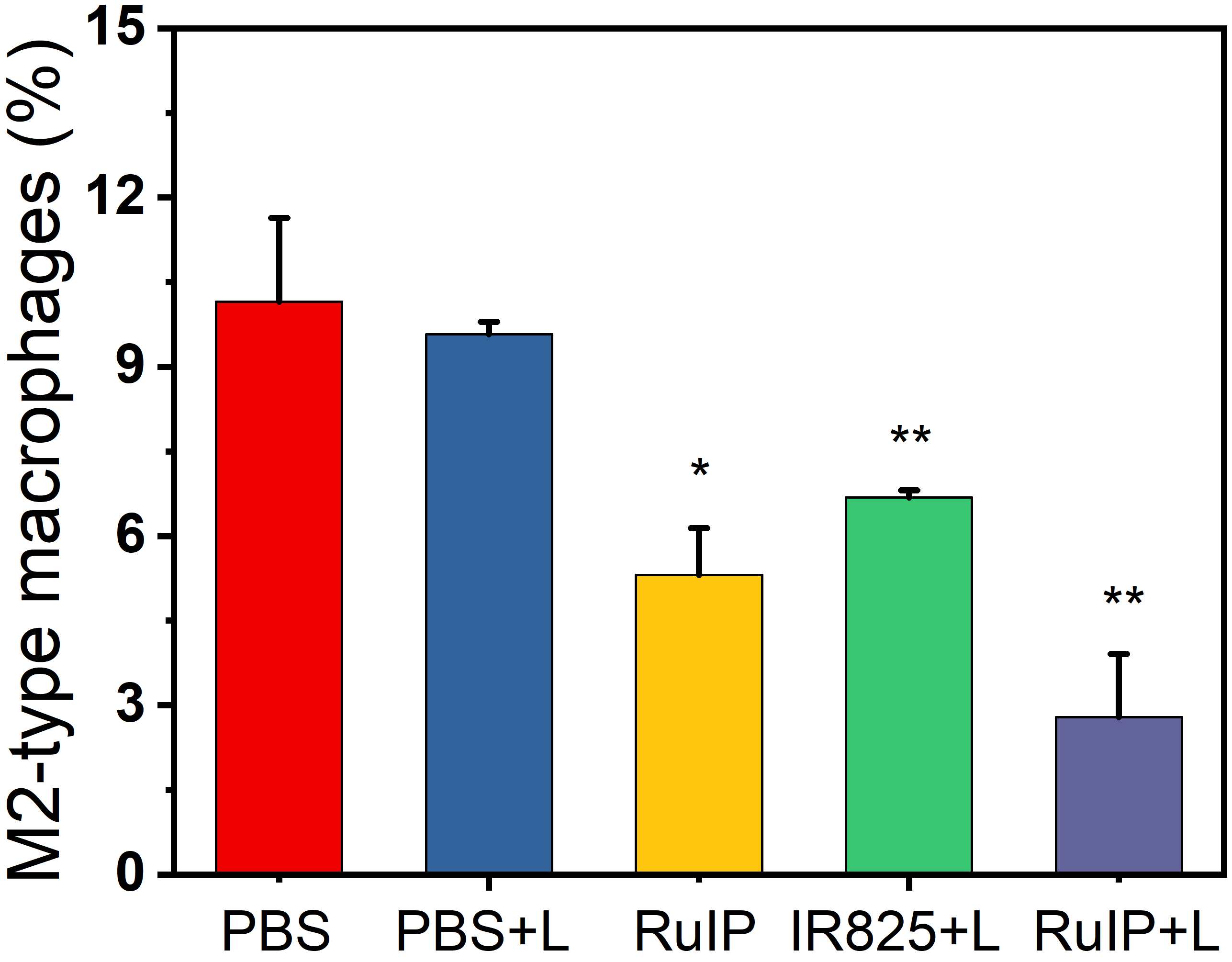


**Figure S19.** Quantitative analysis of cell frequencies of M2 macrophages (n= 3).
